# Ultrastrong MXene films via the synergy of intercalating small flakes and interfacial bridging

**DOI:** 10.1038/s41467-022-35226-0

**Published:** 2022-11-29

**Authors:** Sijie Wan, Xiang Li, Ying Chen, Nana Liu, Shijun Wang, Yi Du, Zhiping Xu, Xuliang Deng, Shixue Dou, Lei Jiang, Qunfeng Cheng

**Affiliations:** 1 School of Chemistry, Key Laboratory of Bio-inspired Smart Interfacial Science and Technology of Ministry of Education, Beihang University, 100191 Beijing, P. R. China; 2 Department of Prosthodontics, The First Clinical Division, Peking University School and Hospital of Stomatology, 100034 Beijing, P. R. China; 3 NMPA Key Laboratory for Dental Materials National Engineering, Laboratory for Digital and Material Technology of Stomatology, Department of Geriatric Dentistry, Peking University School and Hospital of Stomatology, 100081 Beijing, P. R. China; 4 School of Physics, Beihang University, 100191 Beijing, P. R. China; 5 National Center for Nanoscience and Technology, 100190 Beijing, P. R. China; 6 Institute for Superconducting and Electronic Materials, Australian Institute for Innovative Materials, University of Wollongong, Wollongong, NSW 2500 Australia; 7 BUAA-UOW Joint Research Centre, Beihang University, 100191 Beijing, P. R. China; 8 Applied Mechanics Laboratory, Department of Engineering Mechanics and Center for Nano and Micro Mechanics, Tsinghua University, 100084 Beijing, P. R. China; 9 CAS Key Laboratory of Bio-Inspired Materials and Interfacial Science, CAS Center for Excellence in Nanoscience, Technical Institute of Physics and Chemistry, Chinese Academy of Sciences, 100190 Beijing, P. R. China; 10 School of Materials Science and Engineering, Zhengzhou University, 450001 Zhengzhou, P. R. China

**Keywords:** Two-dimensional materials, Mechanical properties

## Abstract

Titanium carbide MXene combines high mechanical and electrical properties and low infrared emissivity, making it of interest for flexible electromagnetic interference (EMI) shielding and thermal camouflage film materials. Conventional wisdom holds that large MXene is the preferable building block to assemble high-performance films. However, the voids in the films comprising large MXene degrade their properties. Although traditional crosslinking strategies can diminish the voids, the electron transport between MXene flakes is usually disrupted by the insulating polymer bonding agents, reducing the electrical conductivity. Here we demonstrate a sequential densification strategy to synergistically remove the voids between MXene flakes while strengthening the interlayer electron transport. Small MXene flakes were first intercalated to fill the voids between multilayer large flakes, followed by interfacial bridging of calcium ions and borate ions to eliminate the remaining voids, including those between monolayer flakes. The obtained MXene films are compact and exhibit high tensile strength (739 MPa), Young’s modulus (72.4 GPa), electrical conductivity (10,336 S cm^−1^), and EMI shielding capacity (71,801 dB cm^2^ g^−1^), as well as excellent oxidation resistance and thermal camouflage performance. The presented strategy provides an avenue for the high-performance assembly of other two-dimensional flakes.

## Abstract (web summary)

Potential application of Titanium carbide MXene in wearable devices is limited by the formation of voids during assembly. Here, the authors demonstrate a synergistic densification strategy by intercalating small flakes and interfacial bridging to obtain high-performance MXene films.

## Introduction

Titanium carbide (Ti_3_C_2_T_x_) MXene^1^ is an emerging two-dimensional transition metal carbide with excellent mechanical properties^2,3^, high electrical conductivity^4^, and low infrared (IR) emissivity^5,6^. Interest in assembling MXene flakes into high-performance macroscopic films has recently grown due to promising applications in flexible electrodes^7–16^, electromagnetic interference (EMI) shielding^17–23^, and thermal camouflage^5,6^, among many others. Because of their higher aspect ratio, large MXene flakes have proven to be better than small ones for making high-performance MXene films^24^. A blade-coating method has been developed to effectively align large MXene flakes^24^, greatly increasing their tensile strength and electrical conductivity. However, the weak interlayer interactions prevent the further improvement of the mechanical properties^25,26^. Moreover, the voids in thick films decrease their properties^27^, thereby limiting many practical applications.

The abundant surface functional groups (T_x_), such as –F, =O, and –OH, allow for chemical crosslinking, including hydrogen^28–30^, ionic^31–33^, and covalent bonding^34–36^, to improve the interfacial strength of adjacent MXene flakes. For example, cellulose nanofiber was embedded into MXene interlayer to reinforce films through hydrogen bonding^29^. MXene-metal ion films were strengthened by ionic bonding^31^. Polydopamine covalently cross-linked with adjacent MXene flakes was used to improve the tensile strength of films^36^. In addition, the combination of hydrogen and covalent bonding agents was recently demonstrated to densify MXene films^27^, resulting in a tensile strength of 583 MPa. While the crosslinking-induced densification strategy can enhance the interlayer interactions and diminish the voids, the use of bonding agents, especially for insulated polymer, usually disrupts the electron transport between MXene flakes and reduces electrical conductivity^27,37^. Thus, it is still challenging to integrate high mechanical and electrical properties into MXene films.

Here, we demonstrate the fabrication of high-performance MXene films using a sequential densification strategy. Small MXene flakes were first intercalated to fill the voids between multilayer large MXene flakes, followed by interfacial bridging of calcium ions (Ca^2+^) and borate ions to synergistically eliminate the remaining voids, including those between monolayer flakes. The resultant sequentially densified MXene (SDM) films integrate high mechanical and electrical properties, as well as excellent oxidation resistance and thermal camouflage.

## Results

### Fabrication of SDM films

Large Ti_3_C_2_T_x_ MXene flakes with an average lateral size of 13.5 μm (Supplementary Fig. 1) were synthesized by selectively etching the Al layer from Ti_3_AlC_2_ MAX phase^38,39^, which was verified by X-ray diffraction (XRD, Supplementary Fig. 2). Small MXene flakes with an average lateral size of 0.35 μm (Supplementary Fig. 3) were obtained by sonicating the as-synthesized MXene dispersion. X-ray photoelectron spectroscopy (XPS, Supplementary Fig. 4) spectra indicate that small MXene flakes have a slightly higher oxygen content than large ones, due to oxidization during sonication^40^. The thickness of both large and small MXene flakes is roughly 1.5 nm (Supplementary Fig. 5), which is larger than the nominal thickness (0.98 nm) of monolayer MXene due to the presence of water on the MXene surface^3^.

**Fig. 1:**
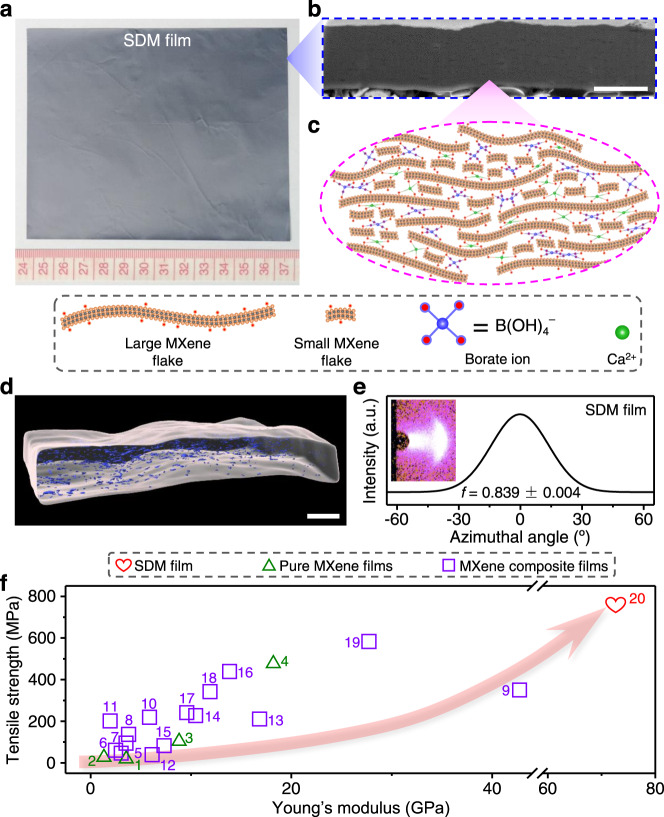
Structural characterization and mechanical performance of SDM films. **a** Photograph showing a lateral size of 13 × 10 cm^2^. **b** SEM image of a cross-section cut by a FIB. Scale bar, 2 μm. **c** Structural model illustrating the intercalation of small flakes and interfacial bridging by Ca^2+^ and borate ions. **d** 3D-reconstructed void microstructure by FIB/SEMT. Scale bar, 2 μm. **e** WAXS pattern for an incident Cu-Kα X-ray beam parallel to the film plane and corresponding azimuthal scan profile for the 002 peak. **f** Tensile strength and Young’s modulus of SDM films (red heart) are shown to exceed those reported for pure MXene films (green triangles) and MXene composite films (purple squares). The sample names, detailed data, and references corresponding to the sample numbers in this plot are in Supplementary Table 6.

The fabrication process of SDM films is shown in Supplementary Fig. 6. Large flake sol was first mixed with small flake sol. The resultant mixture was then doctor blade cast into an intercalation-induced densified MXene (IDM) film. Subsequently, the IDM film was immersed successively into calcium chloride and sodium tetraborate solutions, followed by rinsing with deionized water. Finally, a large-area SDM film (Fig. 1a) was obtained by thermal annealing under a vacuum.

Four kinds of IDM films (IDM-I to IDM-IV) with increasing small flake content were fabricated. The strongest version of the IDM films, corresponding to IDM-II with a small flake content of 10 wt%, was used to fabricate SDM films and compare structure and properties with other types of MXene films. To understand the synergistic effect of inserting small flakes and bridging in this sequential densification strategy, the bridging-induced densified MXene (BDM) films were also fabricated for comparison by treating MXene films comprising large flakes (called LM films) using the same immersing, rinsing, and annealing process. The actual content of Ca^2+^ and boron in SDM and BDM films, derived from XPS, is tabulated in Supplementary Table 1.

### Structural characterization of SDM films

The scanning electron microscopy (SEM) image of the cross-section cut by the focused ion beam (FIB) of the SDM film shows a dense structure (Fig. 1b) with a porosity of 4.11 ± 0.32% (Supplementary Table 2). The structural model of SDM films is shown in Fig. 1c. FIB and SEM tomography (FIB/SEMT, Fig. 1d, Supplementary Fig. 7d, and Supplementary Movies 1 and 2) were used to reconstruct the three-dimensional (3D) void microstructure of SDM films. The volume of 3D-reconstructed voids ranges from 2.5 × 10^−5^ to 6.5 × 10^−2^ μm^3^ (Supplementary Fig. 8d). Since some very small voids having a voxel size lower than dozens of nanometers, such as the voids between monolayer flakes, cannot be detected by FIB/SEMT, the porosities obtained by 3D reconstruction (Supplementary Fig. 7) are lower than those obtained by density measurements (Supplementary Table 2).

Wide-angle X-ray scattering (WAXS) results show that the SDM films have an alignment of 0.839 ± 0.004 (Fig. 1e and Supplementary Fig. 9). XRD curves demonstrate the intercalation of small MXene flakes, Ca^2+^, and borate ions into large MXene interlayers (Supplementary Fig. 10 and Supplementary Table 3). Fourier transform infrared (FTIR, Supplementary Fig. 11) spectra and XPS (Supplementary Fig. 12) confirm the formation of H–O → Ca^2+^ coordination, while XPS, nuclear magnetic resonance (NMR) (Supplementary Fig. 13), and FTIR spectra verify the covalent crosslinking between borate ions and hydroxyl groups on the MXene surface.

### Intercalation-induced densification

**Fig. 2:**
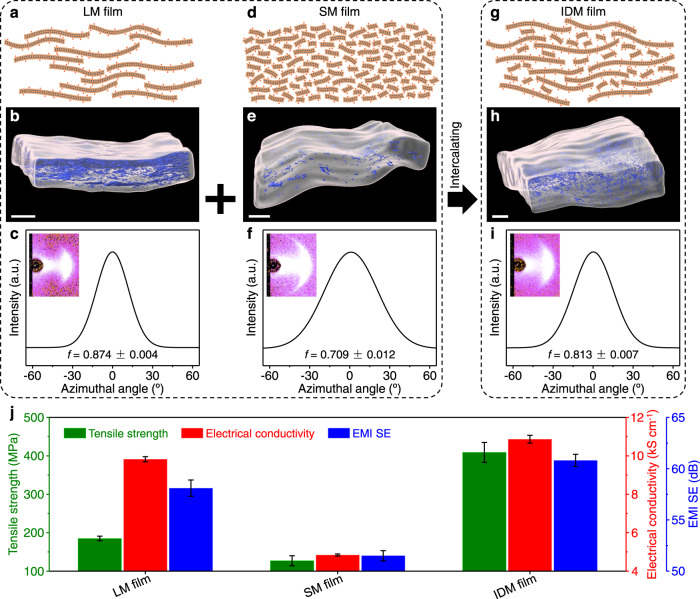
Comparison of structure and properties of LM, SM, and IDM films. **a**–**i** Structural models, 3D-reconstructed void microstructure by FIB/SEMT, and WAXS patterns for an incident Cu-Kα X-ray beam parallel to the film plane and corresponding azimuthal scan profiles for the 002 peak for LM (**a**–**c**), SM (**d**–**f**), and IDM (**g**–**i**) films. The flake alignment of LM, SM, and IDM films is 0.874 ± 0.004, 0.709 ± 0.012, and 0.813 ± 0.007, respectively. Scale bars, 2 μm (**b**, **e**, **h**). **j** Tensile strength, electrical conductivity, and average EMI SE between 0.3 and 18 GHz of LM, SM, and IDM films. EMI SE means electromagnetic interference shielding effectiveness. All error bars show mean ± standard deviation.

Because small flakes with more oxygenated functional groups can absorb more water (Supplementary Fig. 14) and are stacked more disorderly^41,42^, the interplanar spacing of MXene films comprising small flakes (called SM films) is larger than for LM films (Supplementary Table 3). Nevertheless, the porosity of SM films (3.84 ± 0.38%) is lower than for LM films (16.1 ± 0.6%) because LM films have numerous large voids between multilayer flakes^43^. Compared with LM films (Fig. 2a–c and Supplementary Movies 3 and 4), SM films show less oriented yet more dense structure (Fig. 2d–f, Supplementary Fig. 15, and Supplementary Movies 5 and 6). The average volume of 3D-reconstructed voids for SM films is smaller than for LM films (Supplementary Fig. 8).

Intercalating small MXene flakes into large MXene interlayers disrupts the oriented stacking of large flakes (Supplementary Fig. 16) and enlarges the interplanar spacing (Supplementary Table 3), but effectively fills the voids between multilayer flakes and densified the film (Fig. 2g–i and Supplementary Table 2). The flake alignment and film porosity monotonically decrease with the addition of small flakes (Supplementary Fig. 17). An optimized balance between flake alignment and compactness was achieved with a small flake content of 10 wt%. With lower small flake content, the numerous voids between multilayer large flakes impede the load transfer and electron transport, degrading the tensile strength and electrical conductivity. With higher small flake content, the poor flake alignment, agglomeration of small flakes, and increase in defective boundaries also cause degradation in tensile strength and electrical conductivity.

Compared with LM and SM films, the optimized IDM films present a moderately aligned and dense structure (Fig. 2g–i and Supplementary Movies 7 and 8). The tensile strength and electrical conductivity of IDM films are 409 ± 26 MPa and 10,865 ± 203 S cm^−1^ (Fig. 2j and Supplementary Tables 4 and 5), respectively, which are 2.2 and 1.1 times higher than those of LM films (185 ± 6 MPa and 9,822 ± 133 S cm^−1^). In addition, the 2.8-μm-thick IDM films (60.8 ± 0.6 dB, Supplementary Fig. 18) have higher EMI shielding effectiveness (SE) between 0.3 and 18 GHz than the 2.7-μm-thick LM films (58.1 ± 0.8 dB). The small flake intercalation strategy to enhance the properties of MXene films by optimizing the balance between the flake alignment and compactness is different from the conventional approach that uses only large flakes to improve film properties^24^. The intercalation-induced densification strategy can be used to enhance further the properties of previously reported MXene composite films assembled from large flakes.

Theoretical calculation of the flake diffusion shows that a uniform intercalated and compact structure was formed with small flake content lower than 10.2 wt%, while agglomeration occurred with small flake content higher than 10.2 wt% (Supplementary Fig. 19). This result further explains why the optimized properties were achieved with a small flake content of 10 wt%. In addition, the Monte Carlo method was used to simulate the structural evolution of IDM films, and their mechanical properties and interplanar spacing were calculated by the rule of mixtures. The theoretical results (Supplementary Fig. 20) indicate that as small flake content increased, the tensile strength first increased to peak and then decreased, whereas the Young’s modulus and interplanar spacing monotonically increased, which is consistent with experimental results.

### Lap-shear test and fractographic study

**Fig. 3:**
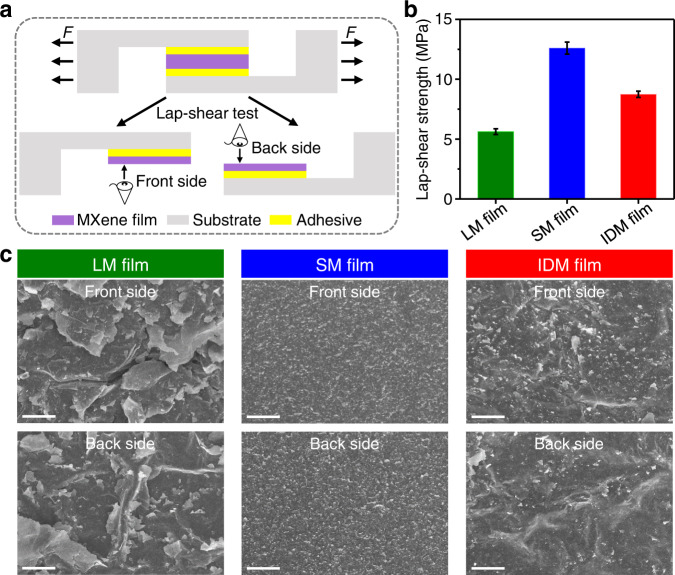
Lap-shear tests of LM, SM, and IDM films. **a** Schematic illustration of the lap-shear test process. A rectangular MXene film with a lateral size of 3 × 4 mm^2^ was glued between two parallel glass substrates using an epoxy adhesive. A shear stress was then applied by pulling the substrates in the opposite direction at a speed of 0.2 mm min^−1^ up to film delamination. After delamination, two fractured surfaces denoted as front and back sides were obtained. **b**, **c** Lap-shear strength (**b**) and SEM images of fractured surfaces (**c**) for LM, SM, and IDM films. Scale bars, 20 μm (**c**). For all tested samples shown here, delamination occurred from within the MXene films, rather than at the adhesive-MXene or adhesive-glass interfaces. All error bars show mean ± standard deviation.

Lap-shear testing^44^ (Fig. 3a) was used to measure the interlayer binding strength of LM, SM, and IDM films. Their lap-shear stress-strain curves are shown in Supplementary Fig. 21. The shear strength of these MXene films decreased as follows: SM > IDM > LM films (Fig. 3b). This is consistent with their porosity, increasing in the following order: SM < IDM < LM films, because voids and defects weaken the interlayer binding^44^.

The front and back sides of the delaminated LM films show large wrinkles (Fig. 3c), which are topologically complementary to each other, suggesting weak interlayer load transfer caused by the voids between multilayer flakes. By contrast, the delaminated surfaces of SM films exhibit ground-glass-like structure with numerous small flakes nearing pull-out, indicating strong interlayer load transfer resulting from dense flake stacking. IDM films present an intermediate morphology with a ground-glass-like structure on the wrinkles, which should be attributed to the intercalation of small flakes that fill the voids between multilayer large flakes to improve interlayer load transfer.

The tensile fracture surface of LM films shows curled edges resulting from the large pull-out of large flakes facilitated by the voids (Supplementary Fig. 22), whereas that of SM films exhibits flat sawtooth-like edges resulting from the restricted slip of small flakes by dense stacking. In addition, the fracture edges of IDM films show intermediate morphology because small flakes fill the voids, preventing the pull-out of large flakes.

### Bridging-induced densification

The interplanar spacing of BDM (1.18 nm, Supplementary Table 3) and SDM (1.21 nm) films is smaller than for LM (1.25 nm) and IDM (1.29 nm) films, respectively, due to a combination of factors, including electrostatic attraction of Ca^2+^, borate crosslinking, and elimination of absorbed water by annealing^27,45–47^. This result indicates that the bridging process diminishes the small voids between monolayer flakes. While the porosity derived from FIB/SEMT for BDM films (4.3%, Supplementary Fig. 7) is lower than for LM films, the BDM films still show some relatively large voids (Supplementary Fig. 23 and Supplementary Movies 9 and 10), indicating that small Ca^2+^ and borate ions cannot effectively bridge the large gap between multilayer flakes. Additionally, BDM and SDM films (Supplementary Fig. 9) have better alignment than do LM and IDM films (Supplementary Fig. 16), demonstrating that the bridging process improves the flake alignment. Compared with LM and IDM films, BDM and SDM films have higher tensile strength and comparable electrical conductivity (Supplementary Tables 4 and 5), because of improved alignment, compactness, and interlayer interactions. In short, this sequential densification strategy presents a synergy that seamlessly integrates the advantages of inserting small flakes and interfacial bridging to effectively eliminate the voids between MXene flakes and strengthen interlayer interactions while maintaining high flake alignment.

### Performance of SDM films

**Fig. 4:**
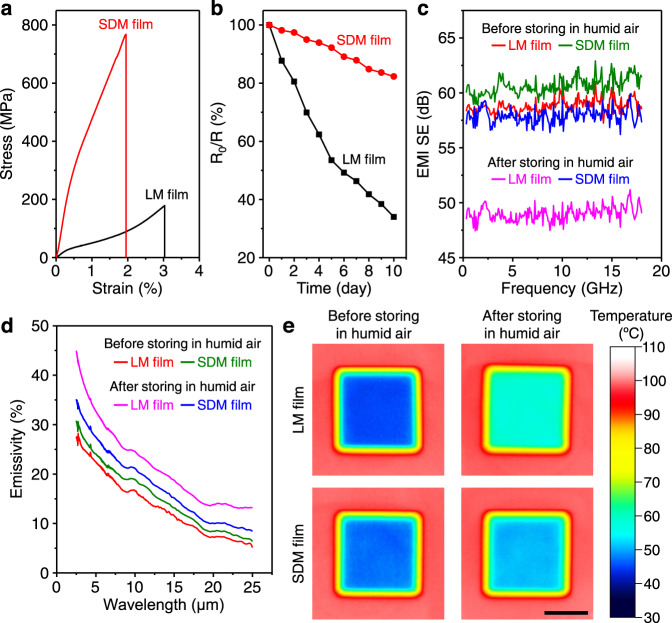
Properties of LM and SDM films. **a** Typical tensile stress-strain curves of as-prepared LM and SDM films. **b** Conductance retention percentages as a function of time for LM and SDM films during 10 days’ storage in humid air with 100% relative humidity. **c**–**e** EMI SE as a function of frequency (**c**), mid-IR emissivity spectra (**d**), and IR photographs on a hot plate with a constant temperature of 100 °C (**e**) for LM and SDM films before and after storage for 10 days in humid air with 100% relative humidity. Scale bar, 1 cm (**e**).

Figure 4a and Supplementary Fig. 24 show tensile stress-strain curves of LM and densified MXene films. Because of the synergistic densification induced by small flake intercalation and interfacial bridging, the SDM films display the highest tensile strength (739 ± 32 MPa, Supplementary Table 4), Young’s modulus (72.4 ± 8.1 GPa), and toughness (8.76 ± 0.52 MJ m^−3^), which are 1.6, 2.4, and 1.5 times higher than those for BDM films; 1.8, 5.3, and 2.1 times higher than those for IDM films; and 4.0, 7.6, and 3.7 times higher than those for LM films, respectively. The tensile strength and Young’s modulus of SDM films surpass those of previously reported MXene films (Fig. 1f). In addition, the SDM films (10,336 ± 103 S cm^−1^, Supplementary Table 5) have higher electrical conductivity than LM films (9822 ± 133 S cm^−1^). The electrical conductivity of SDM films is well above that reported for previous MXene composite films, even exceeding that of some pure MXene films (Supplementary Table 6).

Structural densification can prevent the penetration of oxygen and water into MXene films, slowing their oxidation and thus improving property stability. For example, SDM films have higher conductance retention than LM films during storage in humid air with 100% relative humidity (Fig. 4b). Reflecting excellent electrical conductivity, the 2.5-μm-thick SDM films (59.9 ± 0.6 dB, Fig. 4c) also have higher EMI SE between 0.3 and 18 GHz than 2.7-μm-thick LM films (58.1 ± 0.8 dB). The surface-specific SE of SDM films, defined as the SE divided by thickness and density^48,49^, reaches up to 71,801 dB cm^2^ g^-1^, which exceeds that of most solid shielding materials (Supplementary Table 7). In addition, after storage in humid air for 10 days, the shielding capacity of SDM films was reduced only by 4.34%, which is lower than for LM films (16.2%). The emissivity in the mid-IR band of as-prepared SDM films is slightly higher than for as-prepared LM films, which is probably because the SDM films have poorer flake alignment than the LM films. Moreover, the mid-IR emissivity of SDM films stored in humid air is much lower than for LM films stored in humid air (Fig. 4d), indicating that SDM films have a more stable thermal camouflage performance. As a demonstration, compared with LM films, the surface radiation temperature of SDM films covered on the same substrate increased less after storage in humid air (Fig. 4e).

## Discussion

In summary, here we report a densification strategy to synergistically eliminate the voids between MXene flakes and strengthen interlayer interactions, without disrupting the interlayer electron transport, by sequentially intercalating small flakes and interfacial bridging. The obtained MXene films are highly compact and scalable. They combine high tensile strength, Young’s modulus, toughness, electrical conductivity, EMI shielding capacity, oxidation resistance, and thermal camouflage performance, showing applicability to conditions involving a humid environment and more demanding mechanical loading, such as flexible wearable devices and military stealth cloaks.

## Methods

### Materials

Hydrochloric acid (HCl, 36–38%) was provided by Sinopharm Chemical Reagents Co., Ltd. Lithium fluoride (LiF, ≥99.99%) was purchased from Aladdin. Sodium tetraborate (Na_2_B_4_O_7_, ≥99%) and calcium chloride anhydrous (CaCl_2_, 99%) were received from Adamas-beta. These reagents were not additionally purified before use. Deionized water (DIW, resistivity >18 MΩ cm) was obtained from a Milli-Q Biocel system.

### Preparation of Ti_3_AlC_2_

The pre-alloyed TiAl gas-atomized powder with a size of 74 μm was firstly mixed with TiC powder with a size of 2 μm in a molar ratio of 1:2, and then ball-milled for 24 h in ethanol. Next, the mixture was dried and pressed into a graphite die, followed by sintering for 2 h under an Ar atmosphere at 1500 °C and 30 MPa. Finally, the Ti_3_AlC_2_ powder with a size less than 38 μm was obtained by grinding and sieving the resultant Ti_3_AlC_2_ block.

### Preparation of large and small Ti_3_C_2_T_x_ flakes

Large Ti_3_C_2_T_x_ MXene flakes were prepared from Ti_3_AlC_2_ under Ar flow using a modified minimally intensive layer delamination method^38,39^. LiF (1.6 g) was firstly added into HCl (20 mL, 9 M) in a Teflon reagent bottle and stirred at room temperature for 5 min. Subsequently, one gram of Ti_3_AlC_2_ powder was slowly added and continually stirred at 40 °C for 30 h. After cooling to room temperature, the resulting mixture was centrifuged for 5 min at 1360 × *g* and the supernatant was discarded. The obtained sediment was further washed with DIW by repeating the above centrifugation process 4~5 times until the pH of the supernatant was larger than 5. Next, the swelled sediment was dispersed in DIW and mildly vibrated. The resulting dispersion was then centrifuged at 250 × *g* for 25 min to remove non-exfoliated particles. Finally, the large MXene flakes were collected in the sediment by centrifuging the supernatant at 2260 × *g* for 20 min. Small MXene flakes were prepared by sonicating the as-synthesized large MXene dispersion for 30 min under Ar flow in an ice bath, followed by centrifugation at 2260 × *g* for 30 min to collect the supernatant. The as-prepared MXene dispersion was centrifuged at 16,100 × *g* to obtain a sol having a concentration of ~25 mg mL^−1^ for subsequent experiments.

### Preparation of SDM films

The freshly synthesized large and small MXene flake sols were uniformly mixed by stirring for 15 min and vibrating for 3 min, followed by degassing. The resultant mixture paste was doctor blade cast at a speed of ~3 cm s^−1^ on a flat substrate of Automatic Film Applicator BEVS1811/3 with a blade’s length of ~20 cm and a gap size of ~0.36 mm. Subsequently, the spread paste was dried at 40 °C and peeled from the substrate, obtaining a large-area IDM film. Next, the IDM film was soaked in a pre-prepared CaCl_2_ solution (4 mg mL^−1^) for 12 h and then rinsed five times using DIW to obtain an ionically bridged IDM film (called IB-IDM film). Finally, the IB-IDM film was soaked in a pre-prepared Na_2_B_4_O_7_ solution (4 mg mL^−1^) for 12 h, followed by rinsing five times using DIW and vacuum annealing at 90 °C for 4 h to obtain an SDM film. Based on the addition of small flakes, the following four types of IDM films were prepared: IDM-I (5 wt%), IDM-II (10 wt%), IDM-III (20 wt%), and IDM-IV (40 wt%). Both IB-IDM and SDM films have a small flake content of 10 wt%. The LM and SM films were fabricated by doctor blade casting large flake and small flake sols, respectively. The covalently bridged IDM and LM films (called CB-IDM and CB-LM films) were prepared by treating IDM-II and LM films, respectively, using the same Na_2_B_4_O_7_ soaking, rinsing, and annealing process. Additionally, the ionically bridged LM films (called IB-LM films) were prepared by treating LM films using the same CaCl_2_ soaking and rinsing process, while the BDM films were prepared by treating LM films using the same CaCl_2_ and Na_2_B_4_O_7_ soaking, rinsing, and annealing process.

## Supplementary information

Supplementary Information

Description of Additional Supplementary Files

Supplementary Movie 1

Supplementary Movie 2

Supplementary Movie 3

Supplementary Movie 4

Supplementary Movie 5

Supplementary Movie 6

Supplementary Movie 7

Supplementary Movie 8

Supplementary Movie 9

Supplementary Movie 10

## Data Availability

All the data generated or analyzed during this study have been included in the manuscript and Supplementary Information. All the data are also available from the corresponding author upon request.
