# Supplementary material for: Ultrastrong MXene films via the synergy of intercalating small flakes and interfacial bridging
Source: Nat Commun. 2022 Nov 29;13:7340. doi: 10.1038/s41467-022-35226-0 (PMC9708659; doi:10.1038/s41467-022-35226-0)
Supplement: Supplementary file 1 — Supplementary Information [file 41467_2022_35226_MOESM1_ESM.pdf]

Supplementary Information

Ultrastrong MXene films via the synergy of intercalating small flakes and interfacial bridging

Sijie Wan[1,†], Xiang Li[1,†], Ying Chen[2,3,†], Nana Liu[4,†], Shijun Wang[5], Yi Du[4,6,7], Zhiping Xu[8], Xuliang Deng[3], Shixue Dou[6,7], Lei Jiang[1,7,9], Qunfeng Cheng[1,7,10,*]

[1]School of Chemistry, Key Laboratory of Bio-inspired Smart Interfacial Science and Technology of Ministry of Education, Beihang University, Beijing 100191, P. R. China.
[2]Department of Prosthodontics, The First Clinical Division, Peking University School and Hospital of Stomatology, Beijing 100034, P. R. China.
[3]NMPA Key Laboratory for Dental Materials National Engineering, Laboratory for Digital and Material Technology of Stomatology, Department of Geriatric Dentistry, Peking University School and Hospital of Stomatology, Beijing 100081, P. R. China.
[4]School of Physics, Beihang University, Beijing 100191, P. R. China.
[5]National Center for Nanoscience and Technology, Beijing 100190, P. R. China.
[6]Institute for Superconducting and Electronic Materials, Australian Institute for Innovative Materials, University of Wollongong, Wollongong, New South Wales 2500, Australia.
[7]BUAA-UOW Joint Research Centre, Beihang University, Beijing 100191, P. R. China.
[8]Applied Mechanics Laboratory, Department of Engineering Mechanics and Center for Nano and Micro Mechanics, Tsinghua University, Beijing 100084, P. R. China.
[9]CAS Key Laboratory of Bio-Inspired Materials and Interfacial Science, CAS Center for Excellence in Nanoscience, Technical Institute of Physics and Chemistry, Chinese Academy of Sciences, Beijing 100190, P. R. China.
[10]School of Materials Science and Engineering, Zhengzhou University, Zhengzhou 450001, P. R. China.
[†]These authors contributed equally: Sijie Wan, Xiang Li, Ying Chen, Nana Liu.
[*]To whom correspondence should be addressed. Email: cheng@buaa.edu.cn

This PDF file includes:

Supplementary Methods

Supplementary Figures 1-24

Supplementary Tables 1-7

Supplementary References

Other Supplementary Materials for this manuscript include:

Supplementary Movies 1-10

Supplementary Methods

Characterization: X-ray photoelectron spectroscopy (XPS) spectra were recorded on an ESCALab220i-XL (Thermo Scientific) using a monochromatic Al-Kα X-ray source. ^{11}B nuclear magnetic resonance (NMR) spectra of unannealed and annealed SDM films were measured using a Bruker Avance III 500WB spectrometer. Fourier transform infrared (FTIR) spectra were collected using a Thermo Nicolet NEXUS-470 FTIR instrument in the attenuated total reflection mode. X-ray diffraction (XRD) curves in Supplementary Fig. 2 were obtained on a Shimadzu XRD-6000 using Cu-Kα radiation and a scanning speed of 2° min^{-1}. Thermogravimetric analysis (TGA) curves were recorded under a nitrogen atmosphere using a TG/DTA6300 (NSK, Japan) and a temperature scan rate of 10ºC min^{-1} from room temperature to 600ºC. The water content of films was obtained by the weight loss in 100ºC. Atomic force microscopy (AFM) images of MXene flakes were taken using a Leica TCS SP5 in the tapping mode. Scanning electron microscope (SEM) images were recorded using a field-emission SEM (JEOL-7500F) at an acceleration voltage of 5 kV. Transmission electron microscope (TEM) images were obtained using an FEI Tecnai G20 instrument at 200 kV. The LM, SM, IDM, BDM, and SDM films were cut by a focused ion beam (FIB) to provide cross-sections using a FEI Helios NanoLab 600i with an acceleration voltage of 30 kV and a current decreasing from 230 pA to 40 pA. The serial sections of LM, SM, IDM, BDM, and SDM films were taken by FIB and SEM tomography (FIB/SEMT) having a constant separation of 30 nm. The corresponding three-dimensional (3D) void microstructure was reconstructed using ImageJ.

Wide-angle X-ray scattering (WAXS) tests were conducted on a Xenocs Xeuss SAXS/WAXS System using an incident Cu-Kα X-ray beam parallel to the film plane and striking on the cross-section of the film. The distance between sample and detector is 13.25 cm. The samples for WAXS tests were 1.5-mm-wide, 10-mm-long strips. The scattering patterns were recorded by a PILATUS 300k detector. The XRD curves in Supplementary Fig. 10 were obtained by WAXS measurements. The alignment degree of MXene flakes was quantified by using the Herman's orientation factor (*f*), which is defined as follows[1],

$$f = \frac{1}{2}\left(3\left\langle \cos^2 \phi \right\rangle - 1\right), \tag{1}$$

where $\langle \cos^2\phi \rangle$ is the average value of the square of the cosine of the azimuthal angle for the 002 peak of MXene films, which is calculated as follows,

$$\left\langle \cos^2 \phi \right\rangle = \frac{\int_0^{\pi/2} I(\phi)\cos^2\phi \sin\phi d\phi}{\int_0^{\pi/2} I(\phi)\sin\phi d\phi}, \tag{2}$$

where $I(\phi)$ is the intensity at an azimuthal angle of ϕ.

The volume percent porosity is derived from mass density measurements as follows,

$$V_p = 1 - \frac{\rho}{\rho_0}, \tag{3}$$

where ρ and ρ_0 are the bulk density of MXene films and the theoretical density of MXene films without any voids, respectively. The chemical formula of synthesized large and small $Ti_3C_2T_x$ MXene flakes can be approximately determined to be $Ti_3C_2O_{0.8}(OH)_{0.12}F_{0.28}$ and $Ti_3C_2O_{0.77}(OH)_{0.19}F_{0.27}$ by XPS characterization[2], respectively. It is reasonable to assume that the $Ti_3C_2T_x$ MXene flakes are derived from Ti_3AlC_2 MAX by replacing the Al layer with T_x. Taking the large MXene flakes as an example, the weight ratio of $Ti_3C_2T_x$ MXene and Ti_3AlC_2 MAX for a unit cell can be expressed as 187.9/194.7=0.965 (the molecular weights of $Ti_3C_2O_{0.8}(OH)_{0.12}F_{0.28}$ and Ti_3AlC_2 are 187.9 and 194.7, respectively.), while the volume ratio of $Ti_3C_2T_x$ MXene and Ti_3AlC_2 MAX for a unit cell is equal to their *c*-lattice parameter ratio (20.89/18.62=1.122)[3]. Thus, using the density of Ti_3AlC_2 MAX (4.24 g cm^{-3}: https://m.chemicalbook.com/ProductChemicalPropertiesCB43166219_EN.htm), the theoretical density of large MXene flakes can be expressed as 4.24×0.965/1.122=3.6467 g cm^{-3}. Similarly, the theoretical density of small MXene flakes can be determined to be 3.6580 g cm^{-3}. In our experiments, the MXene films contain intercalated water. Thus, the theoretical densities of LM, SM, IDM, BDM, and SDM films without any voids are obtained based on the theoretical densities of large and small MXene flakes, calcium chloride (2.15 g cm^{-3}), sodium tetraborate (2.367 g cm^{-3}), and water (1 g cm^{-3}), and their composition ratios.

Tensile stress-strain curves were obtained at a strain rate of 1 mm min^{-1} by a Shimadzu AGS-X Tester with a 100 N load cell under a temperature of 25°C and 30% humidity. The samples were adhesively attached to a paper frame with a rectangular hole wide and long enough to accommodate the sample width and gauge length. The gauge length, sample length, and sample width for all MXene films were 5, 10, and 3 mm, respectively. This rigid frame ensured the attachment of the sample to the clamp without damage. The subsequent cutting of the legs of the frame released the sample for tensile tests. The thickness of each tested sample strip was calculated by averaging thickness values at 4 to 6 different positions, which was applied to determine the mechanical properties of the corresponding sample strip. The average mechanical properties and corresponding standard deviations for each sample type were determined based on 10 test results, where the few sample strips that broke near the clamps were excluded from the calculations.

Lap-shear tests of MXene films were performed on a Shimadzu AGS-X Tester with a 1 kN load cell. The MXene films were cut into rectangular sample strips with a lateral size of 3×4 mm^2, which were glued between two parallel glass slides using an epoxy adhesive. Two additional pieces of glass were then glued to the pulling ends of the slides to compensate for the thickness of the slides (See the schematic illustration in Fig. 3a), so that the shear stress is strictly aligned along the in-plane direction of MXene films. Next, two ends of the prepared specimen were gripped and

pulled in opposite directions at a strain rate of 0.2 mm min^{-1} up to film delamination. After all the tests, the fracture surfaces were examined by SEM to ensure the same delamination from within MXene films, rather than from the interface of MXene/adhesive or adhesive/glass. Shear stress was calculated by dividing the recorded shear force by the area of sample (12 mm^2). Values of shear strength were obtained by the stress upon delamination.

Electrical conductivities and resistances were measured using a two-probe method on a Keithley 2400 multiple-function source meter. The samples applied for electrical conductivity and resistance tests were 2-mm-wide, 30-mm-long strips. Two probes were connected with the two ends of sample strips by silver paste. The average electrical conductivity and corresponding standard deviation for each sample type were determined based on 3 to 5 test results.

Electromagnetic interference (EMI) shielding capacities were tested in the frequency range between 0.3 and 18 GHz by a coaxial air-line method using an AV3629 vector network analyzer. The samples applied for EMI shielding measurements were circular films with a diameter of 10 mm. Scattering parameters were recorded to determine the EMI shielding effectiveness (SE)[4]. The average values and standard deviation of the EMI SE for each sample type were determined based on 3 to 5 test results.

Theoretical calculation of the flake diffusion. It is reasonable to assume that the small MXene flakes are randomly distributed among large ones at the initial stage (Supplementary Fig. 19a). The mass ratio of small flakes to large ones is expressed as follows,

$$\frac{\phi}{1-\phi}=\frac{n_{\mathrm{s}}\rho_{\mathrm{s}}l_{\mathrm{s}}^{2}}{n_{\mathrm{L}}\rho_{\mathrm{L}}l_{\mathrm{L}}^{2}}, \tag{4}$$

where ϕ, n_s, l_s, and ρ_s are the mass fraction, number, lateral size, and density of small flakes, respectively, while n_L, l_L, and ρ_L are the number, lateral size, and density of large flakes. The ρ_s/ρ_L is approximately equal to 1. Thus, the average number (n) of small flakes on a large flake is given as follows,

$$n=\frac{n_{\mathrm{s}}}{n_{\mathrm{L}}}=\left(\frac{l_{\mathrm{L}}}{l_{\mathrm{s}}}\right)^{2}\frac{\phi}{1-\phi}, \tag{5}$$

The average distance between small flakes at the initial stage for the doctor blade casting is determined to be $d=l_L/n^{0.5}$ (Supplementary Fig. 19b). The average diffusion distance of small flakes in the subsequent drying process is $d_t=(D_t t_0)^{0.5}$, where D_t and t_0 are the translational diffusion coefficient and diffusion time of small flakes, respectively. The condition for uniformly distributing the small flakes is $d \geq d_t$. Thus, combined with equation (5), the critical mass fraction (ϕ_{cr}) for the uniform distribution of small flakes is given as follows,

$$\phi_{\mathrm{cr}}=\frac{l_{\mathrm{s}}^{2}}{D_{\mathrm{t}}t_{0}+l_{\mathrm{s}}^{2}}, \tag{6}$$

Below ϕ_{cr}, the small flakes are uniformly intercalated into large MXene interlayers (Supplementary Fig. 19c) to enhance the tensile strength of IDM films, whereas above ϕ_{cr}, the small flakes agglomerate at the gaps between large flakes (Supplementary Fig. 19d), decreasing the tensile strength. The typical diffusion time during drying is $t_0=t_d/N$, where t_d is the drying time (~10 min) before gelation and N is the layer number (~3061) of obtained IDM films with a thickness of ~3 μm. Using a disk model in lateral motion, the D_t is given as follows[5],

$$D_t = \frac{k_B T \ln\left(\frac{l_s}{b}\right)}{4\pi\eta l_s}, \quad (7)$$

where l_s is the lateral size (0.35 μm) of small flakes, b is the theoretical thickness (0.98 nm) of small flakes[6], η is the viscosity (1.0 mPa·s) of water, k_B is Boltzmann constant (1.380649×10^{-23} J K^{-1}), and T is room temperature (298.15 K). Substituting D_t (5.50×10^{-12} m^2 s^{-1}) into equation (6), the ϕ_{cr} is determined to be 10.2 wt%, which is close to the mass fraction (10 wt%) of small flakes for the strongest IDM films in our experiments. This result provides a qualitative explanation for the optimization of the tensile strength of IDM films.

Theoretical modeling of the structural evolution, mechanical properties, and interplanar spacing of IDM films. In the above theoretical simulation, the final dispersion state of small flakes in IDM films was ideally simplified by comparing only the diffusion distance of small flakes with their initial spacing. More realistically, the intercalation and agglomeration zones might coexist in IDM films. Here, the Monte Carlo method was used to simulate the diffusion process of small MXene flakes among large ones and study the structural evolution of IDM films in the casting and drying process. The small flakes are randomly distributed among large ones at the initial stage. The length of the gap between large flakes, l_{gap}, is estimated by the difference between the porosities of LM (16.1%) and SM (3.84%) films. The potential energy of small flakes is determined by their positions. In the uniform intercalation zone, the small flakes move freely, and the corresponding potential energy is a constant, $E_1=E_0$. In the small flake agglomeration zone, the potential energy is defined as $E_2=E_0[1–\exp(–\alpha\phi_2/(1–\phi))]$, where ϕ_2 is the ratio of the mass for small flakes in the agglomeration zone to total mass and α is a constant. ϕ_1 is the ratio of the mass for small flakes in the intercalation zone to total mass and meets $\phi=\phi_1+\phi_2$. At each step, each small flake is assigned a trial move $–\Delta l_{max}<\Delta l<\Delta l_{max}$, and the corresponding energy difference, ΔE, is calculated. The trial move is accepted or rejected according to the Metropolis algorithm[7]: (1) $\Delta E\leq0$ always accepted, and (2) $\Delta E>0$ accepted with a probability of $\exp(–\Delta E/k_BT)$. The dimensionless parameters used in the Monte Carlo model are shown in Supplementary Fig. 19e.

The ϕ_1 and ϕ_2 evolve with the time and eventually reach a thermodynamic equilibrium (Supplementary Fig. 19f). The ratio of energy barrier to thermal fluctuation energy, E_0/k_BT, has impacts on the diffusion kinetics of small flakes and the final equilibrium structure of IDM films.

In our simulation, the E_0/k_BT is set as 2, which can give a good prediction for the mechanical properties and interplanar spacing of IDM films. Using the rule of mixtures based on the intercalation zone and agglomeration zone, the tensile strength (σ_{IDM}), Young's modulus (E_{IDM}), and average interplanar spacing (h_{IDM}) of IDM films are simulated as follows,

$$\sigma_{\mathrm{IDM}} = \left(1-\phi_2\right)\sigma_{\mathrm{IZ}} + \phi_2\sigma_{\mathrm{SM}}, \tag{8}$$

$$E_{\mathrm{IDM}} = \left(1-\phi_2\right)E_{\mathrm{IZ}} + \phi_2 E_{\mathrm{SM}}, \tag{9}$$

$$h_{\mathrm{IDM}} = \left(1-\phi_2\right)h_{\mathrm{IZ}} + \phi_2 h_{\mathrm{SM}}, \tag{10}$$

where σ_{SM}, E_{SM}, and h_{SM} are the tensile strength, Young's modulus, and interplanar spacing of SM films (agglomeration zone), while σ_{IZ}, E_{IZ}, and h_{IZ} are the tensile strength, Young's modulus, and interplanar spacing of the intercalation zone, respectively. The experimental results indicate that the intercalation of small flakes into large MXene interlayers caused the decrease in the porosity and alignment. As a result, the strength of IDM films reached a peak with a small flake content of 10 wt%, while the Young's modulus and interplanar spacing monotonically increased with the small flake content. Here we assume that there also exists an optimal mass ratio (β) of small flakes to large ones in the ideal intercalation zone to optimize its strength. The corresponding tensile strength, Young's modulus, and interplanar spacing are σ_β, E_β, and h_β, respectively. The σ_{IZ}, E_{IZ}, and h_{IZ} are then given as follows,

$$\sigma_{\mathrm{IZ}} = \begin{cases} \sigma_{\mathrm{LM}} + \left(\sigma_\beta - \sigma_{\mathrm{LM}}\right)\dfrac{\phi_1}{\beta\phi_{\mathrm{L}}}, & \dfrac{\phi_1}{\phi_{\mathrm{L}}} \le \beta \\ \sigma_\beta + \left(\sigma_{\mathrm{SM}} - \sigma_\beta\right)\dfrac{\Delta\phi_1}{\phi_1}, & \dfrac{\phi_1}{\phi_{\mathrm{L}}} > \beta \end{cases}, \tag{11}$$

$$E_{\mathrm{IZ}} = \begin{cases} E_{\mathrm{LM}} + \left(E_\beta - E_{\mathrm{LM}}\right)\dfrac{\phi_1}{\beta\phi_{\mathrm{L}}}, & \dfrac{\phi_1}{\phi_{\mathrm{L}}} \le \beta \\ E_\beta\left(1-\dfrac{\Delta\phi_1}{1-\phi_2}\right) + E_{\mathrm{SM}}\dfrac{\Delta\phi_1}{1-\phi_2}, & \dfrac{\phi_1}{\phi_{\mathrm{L}}} > \beta \end{cases}, \tag{12}$$

$$h_{\mathrm{IZ}} = \begin{cases} h_{\mathrm{LM}} + \left(h_\beta - h_{\mathrm{LM}}\right)\dfrac{\phi_1}{\beta\phi_{\mathrm{L}}}, & \dfrac{\phi_1}{\phi_{\mathrm{L}}} \le \beta \\ h_\beta\left(1-\dfrac{\Delta\phi_1}{1-\phi_2}\right) + h_{\mathrm{SM}}\dfrac{\Delta\phi_1}{1-\phi_2}, & \dfrac{\phi_1}{\phi_{\mathrm{L}}} > \beta \end{cases}, \tag{13}$$

where σ_{LM}, E_{LM}, and h_{LM} are the tensile strength, Young's modulus, and interplanar spacing of LM films, respectively, ϕ_L is the mass fraction of large MXene flakes, and $\Delta\phi_1=\phi_1-\beta\phi_L$. Here β, σ_β, E_β, and h_β are set as 0.065, 470 MPa, 13.0 GPa, and 1.30 nm, respectively, which can provide a good prediction for the mechanical properties and interplanar spacing of IDM films (Supplementary Fig. 20).

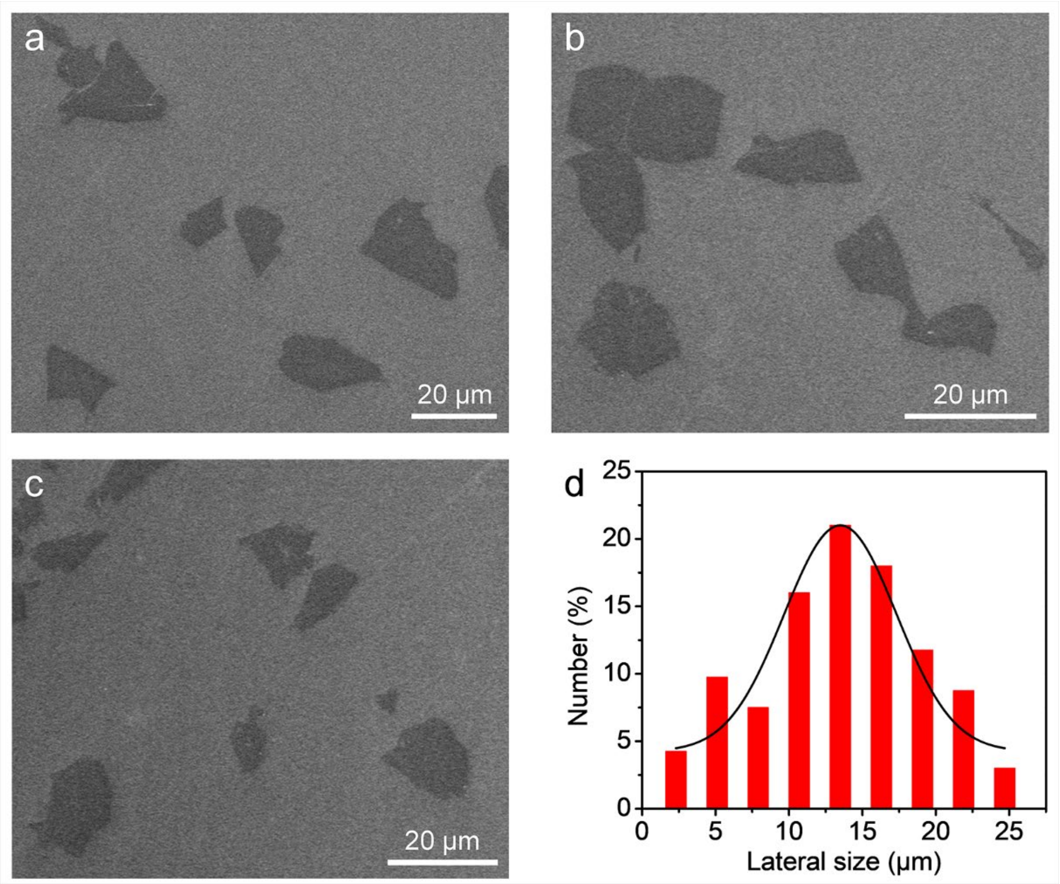

Supplementary Figure 1. Calculation of lateral size of large MXene flakes.
a–**c**, SEM images of large MXene flakes. **d**, Lateral size distribution of large MXene flakes, indicating that the average lateral size of large MXene flakes is roughly 13.5 µm.

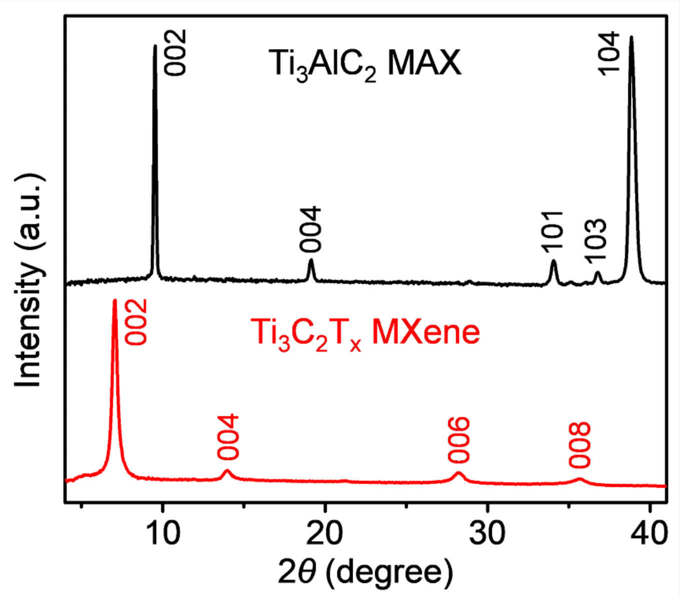

Supplementary Figure 2. XRD curves using Cu-Kα radiation for Ti_3AlC_2 MAX and $Ti_3C_2T_x$ MXene.

The 104 peak of Ti_3AlC_2 MAX at ~39º is absent in the curve of $Ti_3C_2T_x$ MXene, demonstrating the successful exfoliation of MXene flakes[8]. Additionally, due to the introduction of terminal functional groups and absorbed water[8,9], the interplanar spacing (002) of $Ti_3C_2T_x$ MXene (1.25 nm, Supplementary Table 3) is much larger than for Ti_3AlC_2 MAX (0.93 nm).

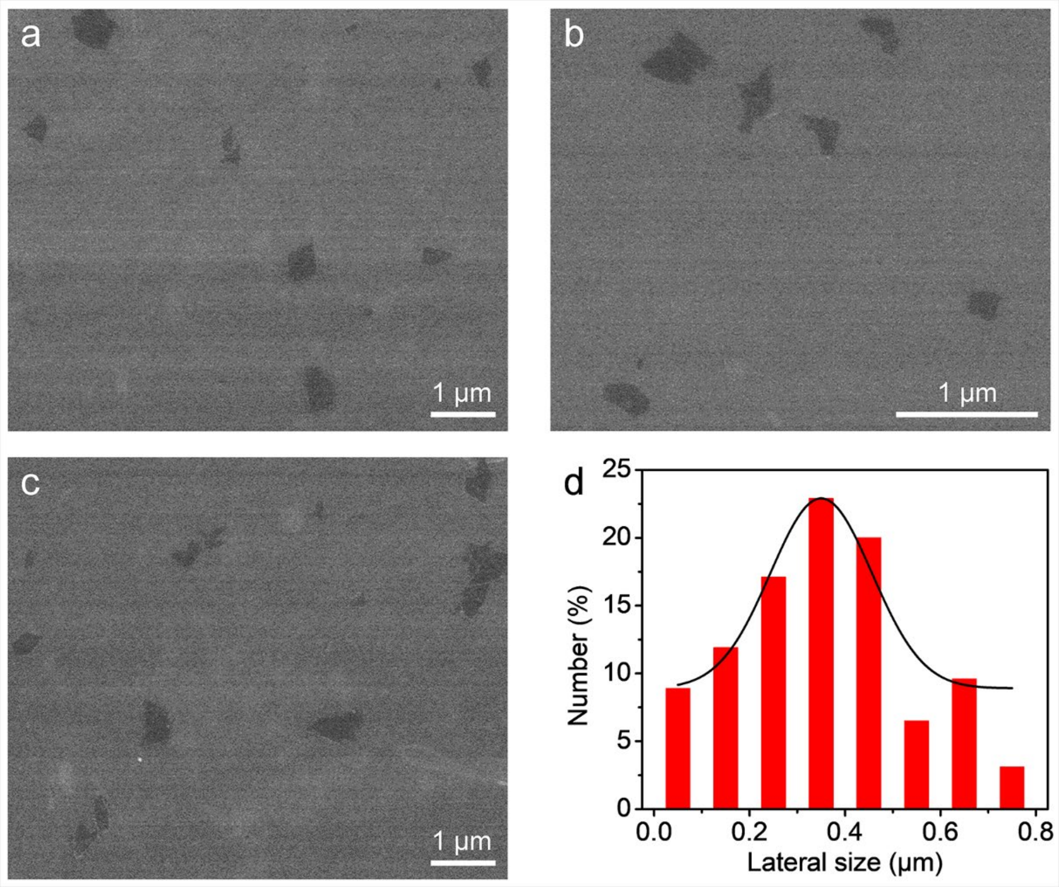

Supplementary Figure 3. Calculation of lateral size of small MXene flakes.
a–**c**, SEM images of small MXene flakes. **d**, Lateral size distribution of small MXene flakes, indicating that the average lateral size of small MXene flakes is roughly 0.35 µm.

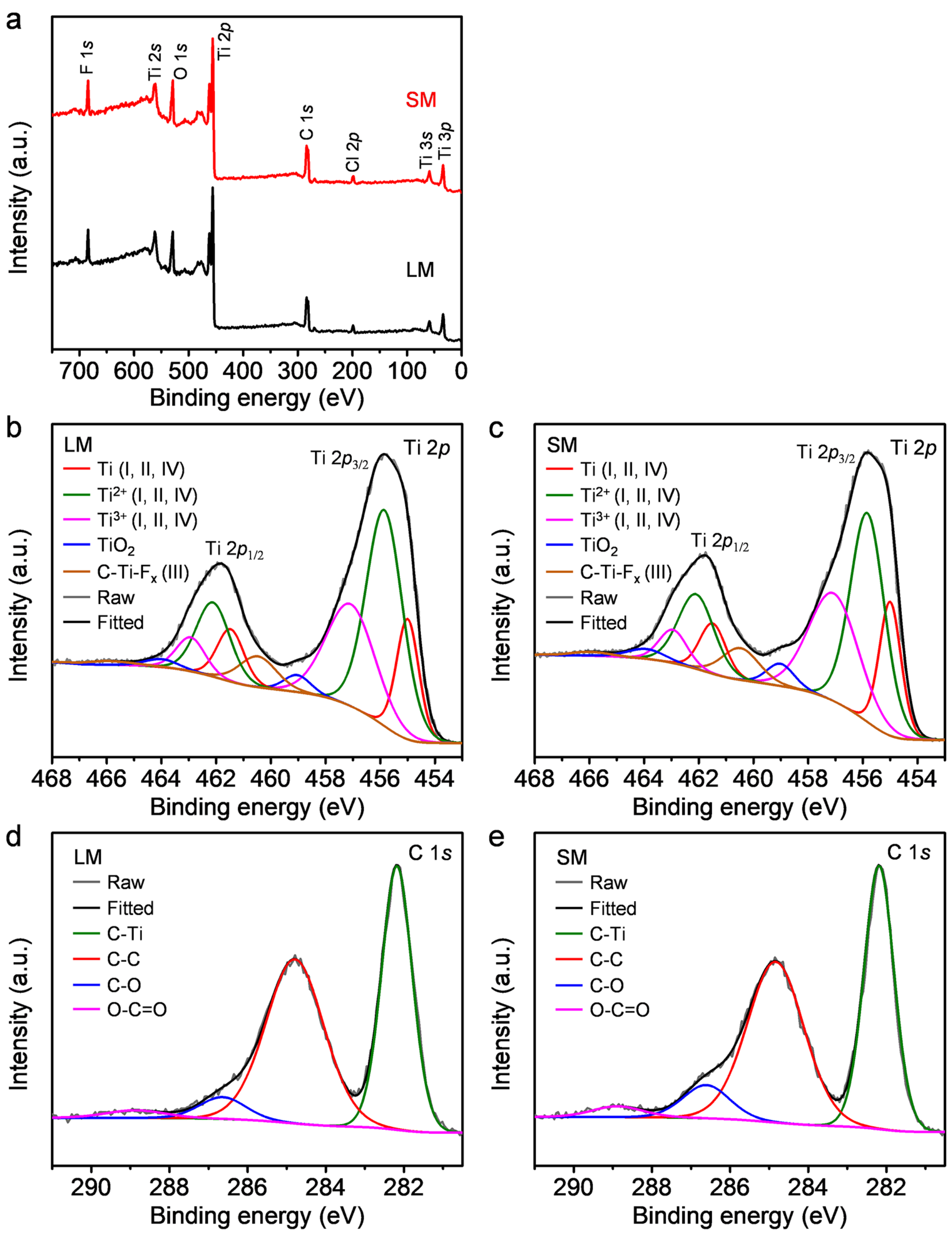

Supplementary Figure 4. XPS spectra characterization of LM and SM films.

a, XPS spectra of LM and SM films. **b**, **c**, Ti 2*p* spectra of LM (**b**) and SM (**c**) films. **d**, **e**, C 1*s* spectra of LM (**d**) and SM (**e**) films. The Ti $2p_{3/2}$ main peak of LM and SM films can be fitted by five peaks at 455.0 (Ti (I, II, IV)), 455.8 (Ti^{2+} (I, II, IV)), 457.1 (Ti^{3+} (I, II, IV)), 459.0 (TiO_2), and 460.5 (C-Ti–F_x (III)) eV, while the C 1*s* peak of LM and SM films can be fitted by four peaks at 282.2 (C−Ti), 284.8 (C−C), 286.6 (C−O), and 288.9 (O−C=O) eV. The TiO_2, C−O, and O−C=O peaks of SM films are a little larger than those of LM films, suggesting that slight oxidization occurred in the fabrication process of small flakes (by sonicating the large flakes)[10].

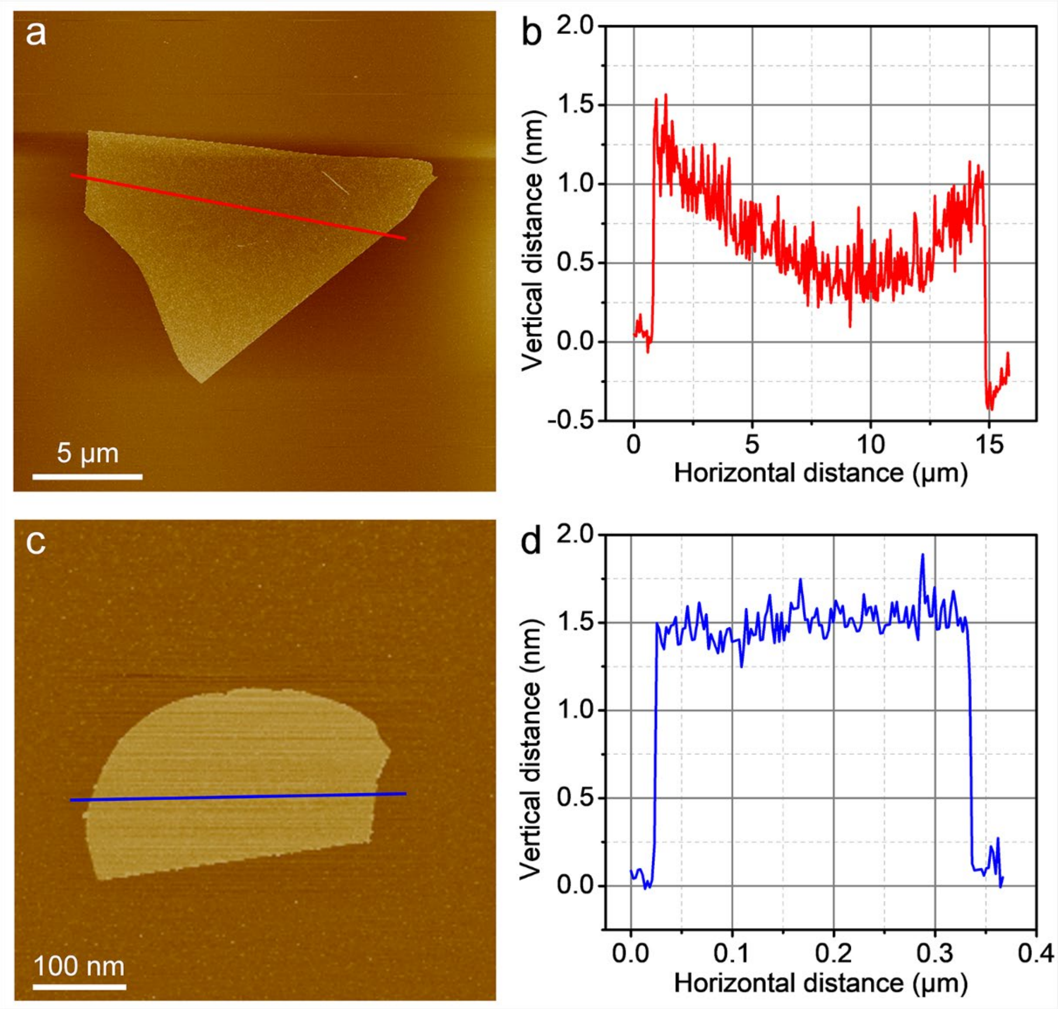

Supplementary Figure 5. Thickness of large and small MXene flakes.
a, **c**, AFM images of large (**a**) and small (**c**) MXene flakes. **b**, **d**, Height profiles for large MXene flakes (**b**) in **a** measured along the red line and small MXene flakes (**d**) in **c** measured along the blue line. Both large and small MXene flakes have a thickness of 1.5 nm, indicating a monolayer structure[11].

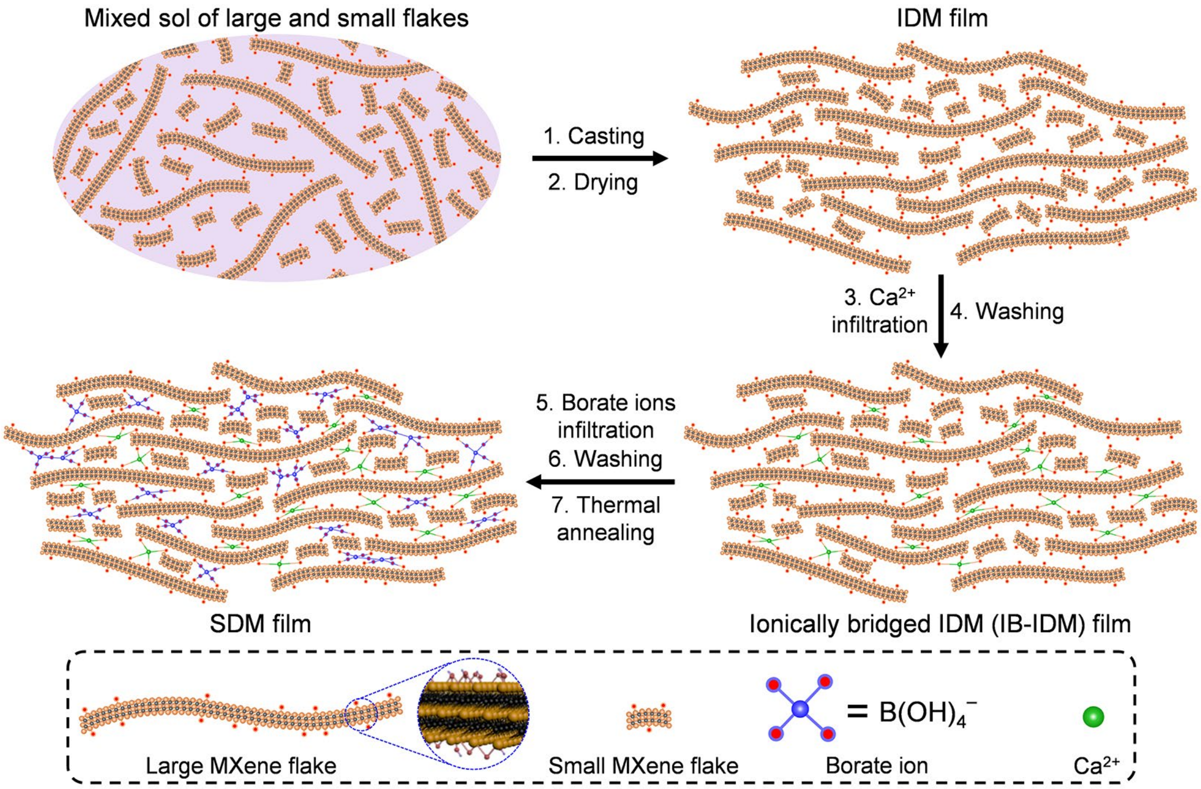

Supplementary Figure 6. Schematic illustration for the fabrication process of SDM films. The as-synthesized large and small MXene flake sols were uniformly mixed and then doctor blade cast on a flat Teflon substrate. After drying, a large-area IDM film was peeled from the substrate. Subsequently, the IDM film was immersed into $CaCl_2$ solution and then rinsed to provide an ionically bridged IDM film (called IB-IDM film). Finally, the IB-IDM film was immersed into $Na_2B_4O_7$ solution, followed by rinsing and vacuum annealing to provide an SDM film.

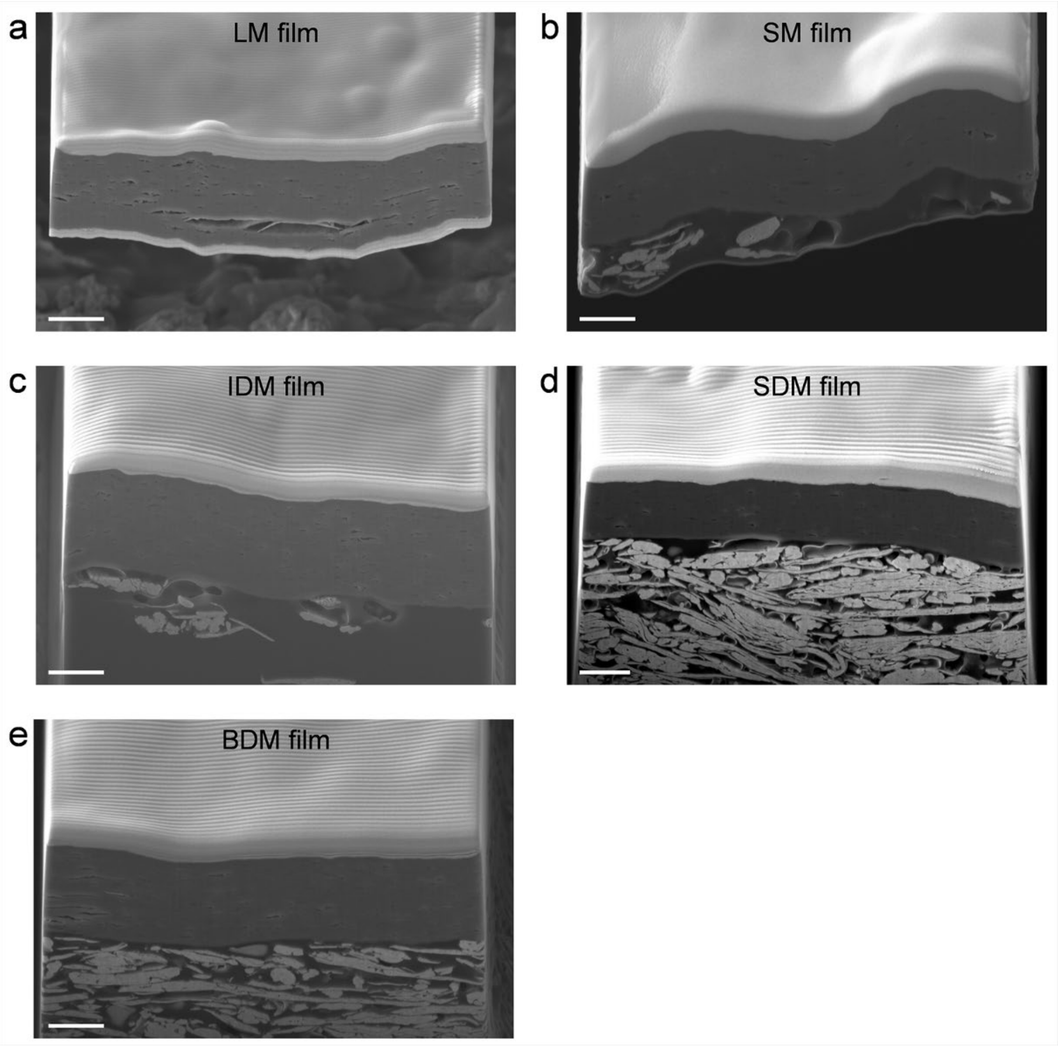

Supplementary Figure 7. FIB/SEMT cross-sections of the films.

a, LM. **b**, SM. **c**, IDM. **d**, SDM. **e**, BDM. Scale bars, 2 μm. Note that the serial section derived from FIB/SEMT is not perpendicular to the film surface. The porosities derived from FIB/SEMT for LM, SM, IDM, SDM, and BDM films are 7.5%, 0.88%, 3.1%, 0.97%, and 4.3%, respectively.

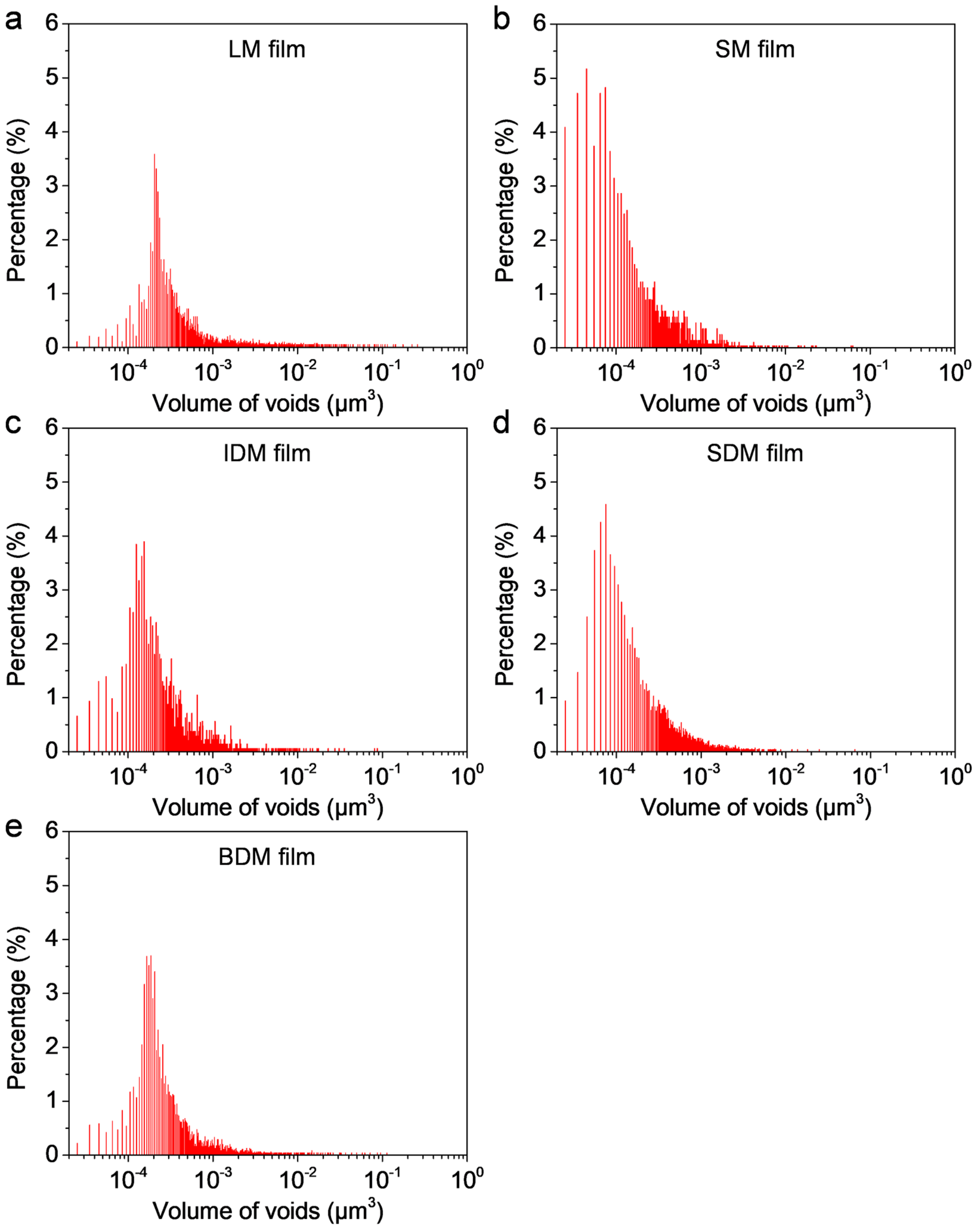

Supplementary Figure 8. Volume distribution of 3D-reconstructed voids derived from FIB/SEMT for films.

a, LM. **b**, SM. **c**, IDM. **d**, SDM. **e**, BDM. The volume ranges for the 3D-reconstructed voids of LM, SM, IDM, SDM, and BDM films are 2.5×10^{-5} to 2.6×10^{-1} μm^3, 2.5×10^{-5} to 6.3×10^{-2} μm^3, 2.5×10^{-5} to 8.7×10^{-2} μm^3, 2.5×10^{-5} to 6.5×10^{-2} μm^3, and 2.5×10^{-5} to 1.1×10^{-1} μm^3, respectively. Note that some very small voids, with a voxel size of dozens of nanometers, could not be observed by FIB/SEMT.

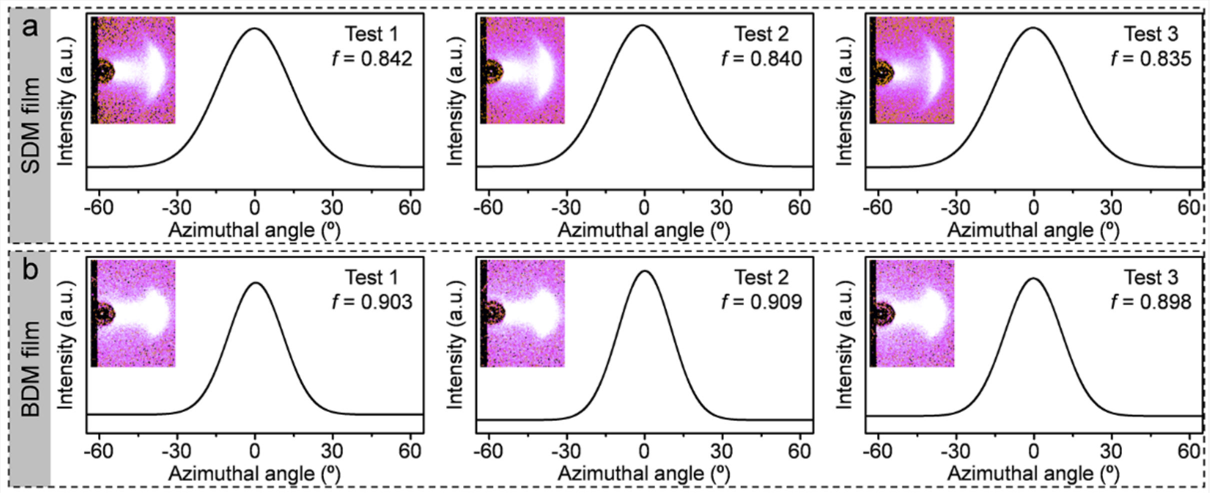

Supplementary Figure 9. WAXS patterns for an incident Cu-Kα X-ray beam parallel to the film plane and corresponding azimuthal scan profiles for the 002 peak for films. a, SDM. **b**, BDM.

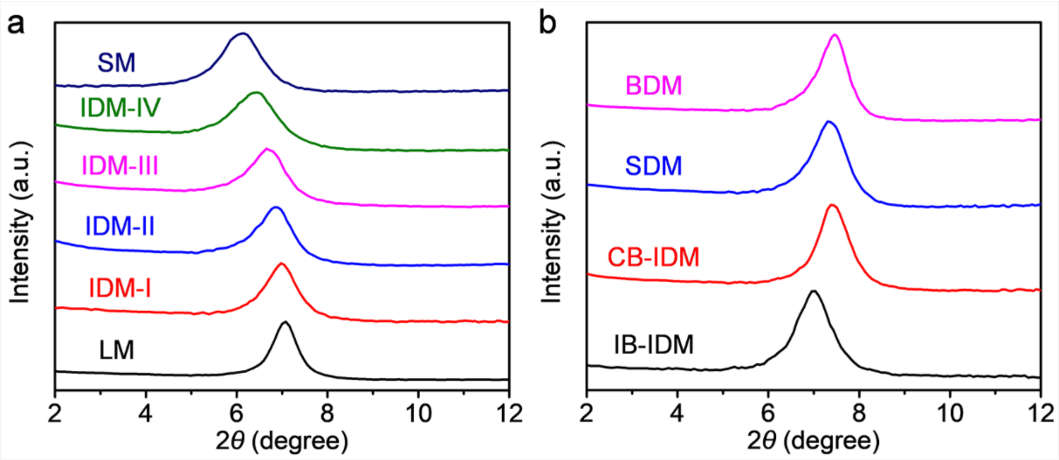

Supplementary Figure 10. XRD curves using Cu-Kα radiation.

a, LM, SM, and IDM films. **b**, IB-IDM, CB-IDM, BDM, and SDM films. Due to more absorbed water and a more disordered structure, the interplanar spacing of SM films is larger than for LM films. The IDM films also have a larger interplanar spacing than LM films, indicating the intercalation of small flakes into large MXene interlayers. The electrostatic attraction of Ca^{2+} slightly decreased the interplanar spacing of IB-IDM films[12]. Additionally, due to borate crosslinking[13] and the elimination of absorbed water by annealing[14], the CB-IDM films have a shorter interplanar spacing than the IDM films. The interplanar spacing of BDM and SDM films is shorter than for LM and IDM films, respectively, demonstrating the insertion of Ca^{2+} and borate ions into MXene interlayers.

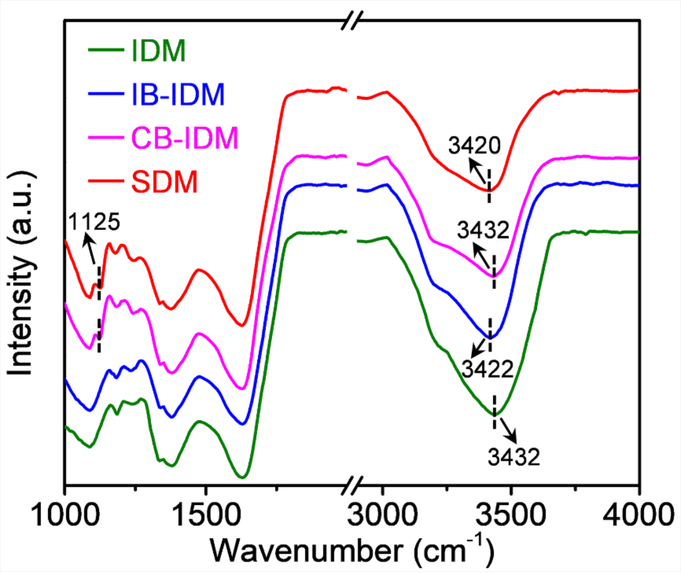

Supplementary Figure 11. FTIR spectra of IDM, IB-IDM, CB-IDM, and SDM films.

The –OH peak is redshifted from 3,432 cm^{-1} for IDM films to 3,422 cm^{-1} for IB-IDM films, indicating the formation of H–O→Ca^{2+} coordination[15]. Additionally, compared with IDM films, the CB-IDM films have a weaker –OH peak and a new B-O peak (1125 cm^{-1}), suggesting the covalent crosslinking between borate ions and –OH groups on the MXene surface[8,16,17]. These peak changes can also be observed in SDM films, confirming the ionic and covalent bonding in SDM films.

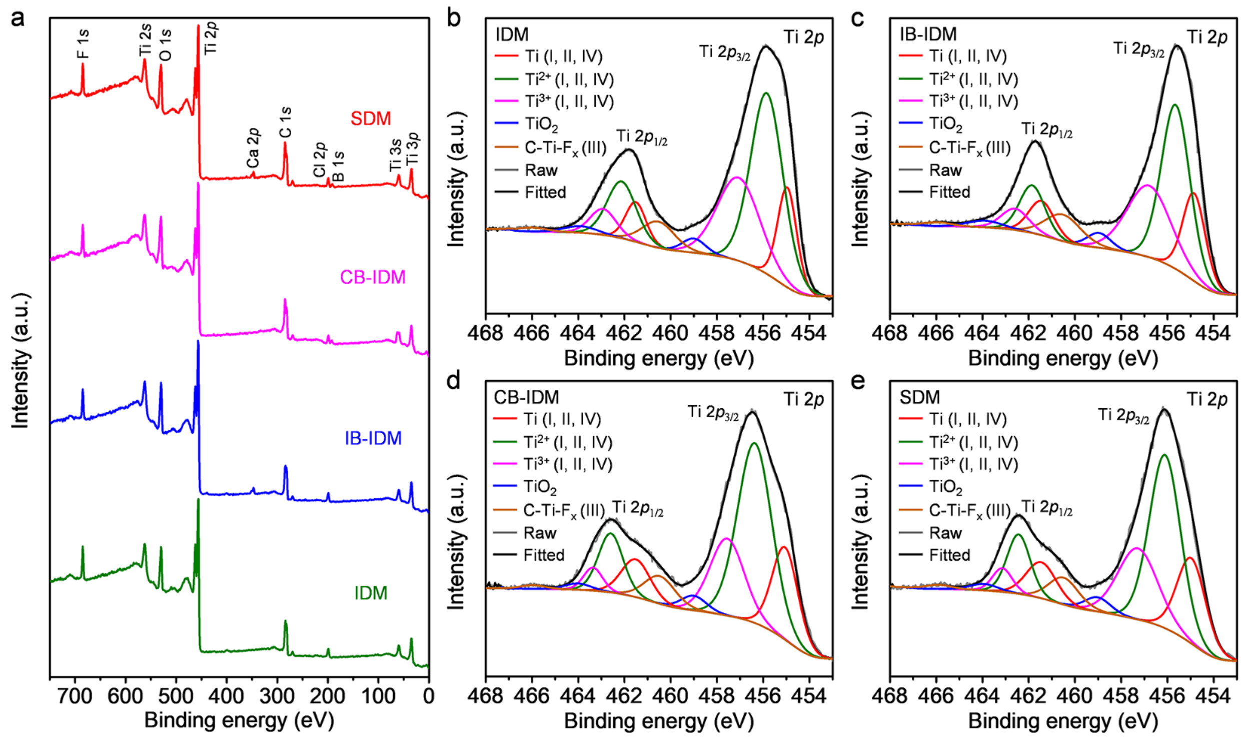

Supplementary Figure 12. XPS spectra characterization of IDM, IB-IDM, CB-IDM, and SDM films.

a, XPS spectra of IDM, IB-IDM, CB-IDM, and SDM films. **b**–**e**, Ti 2*p* spectra of IDM (**b**), IB-IDM (**c**), CB-IDM (**d**), and SDM (**e**) films. Compared with IDM films, the SDM films have new Ca 2*p* and B 1*s* peaks, indicating the modification of Ca^{2+} and borate ions. The Ti $2p_{3/2}$ main peak of IDM films can be fitted by five peaks at 455.0 (Ti (I, II, IV)), 455.8 (Ti^{2+} (I, II, IV)), 457.1 (Ti^{3+} (I, II, IV)), 459.0 (TiO_2), and 460.5 (C-Ti–F_x (III)) eV. The Ti $2p_{3/2}$ main peak of IB-IDM films can be fitted by five peaks at 455.0 (Ti (I, II, IV)), 455.6 (Ti^{2+} (I, II, IV)), 456.8 (Ti^{3+} (I, II, IV)), 459.0 (TiO_2), and 460.5 (C-Ti–F_x (III)) eV. The Ti $2p_{3/2}$ main peak of CB-IDM films can be fitted by five peaks at 455.0 (Ti (I, II, IV)), 456.4 (Ti^{2+} (I, II, IV)), 457.6 (Ti^{3+} (I, II, IV)), 459.0 (TiO_2), and 460.5 (C-Ti–F_x (III)) eV. The Ti $2p_{3/2}$ main peak of SDM films can be fitted by five peaks at 455.0 (Ti (I, II, IV)), 456.1 (Ti^{2+} (I, II, IV)), 457.3 (Ti^{3+} (I, II, IV)), 459.0 (TiO_2), and 460.5 (C-Ti–F_x (III)) eV. The Ti^{2+} (I, II, IV) $2p_{3/2}$ and Ti^{3+} (I, II, IV) $2p_{3/2}$ peaks are downshifted from 455.8 and 457.1 eV for IDM films to 455.6 and 456.8 eV for IB-IDM films, respectively. This is because the Ca atoms ionically bonded onto MXene nanosheets have lower electronegativity than do Ti atoms, increasing the electron cloud density of Ti atoms[9]. By contrast, the Ti^{2+} (I, II, IV) $2p_{3/2}$ and Ti^{3+} (I, II, IV) $2p_{3/2}$ peaks are upshifted from 455.8 and 457.1 eV for IDM films to 456.4 and 457.6 eV for CB-IDM films, respectively. This is because the boron atoms having higher electronegativity than Ti atoms are covalently bonded with Ti atoms by borate orthoester bonds and decrease the electron cloud density of Ti atoms[8]. These peak changes can also be observed in SDM films, verifying the ionic and covalent bonding in SDM films.

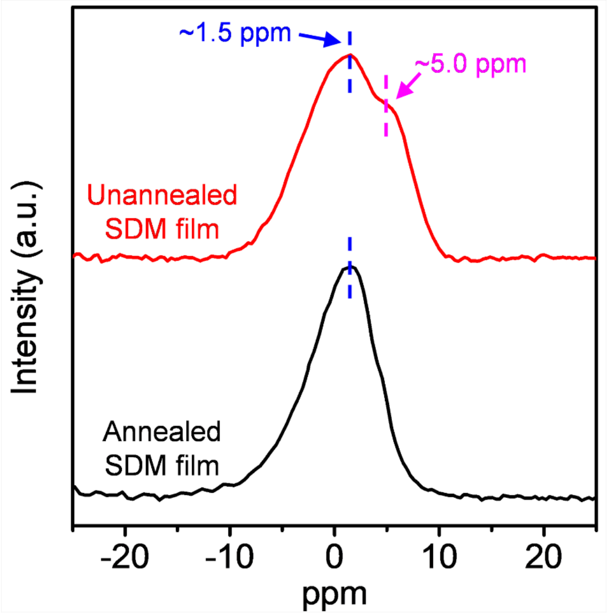

Supplementary Figure 13. ^{11}B NMR spectra of unannealed and annealed SDM films.
The ^{11}B NMR spectrum of annealed SDM films shows a distinct peak at ~1.5 ppm corresponding to the fully esterified borate orthoesters[16], verifying the successful reaction between borate ions and –OH groups on the surface of MXene flakes. Additionally, the peak at ~5.0 ppm of unannealed SDM films, corresponding to the partially esterified borate orthoesters[16], almost disappears in the annealed SDM films, indicating that annealing drives the formation of many more borate orthoester bonds.

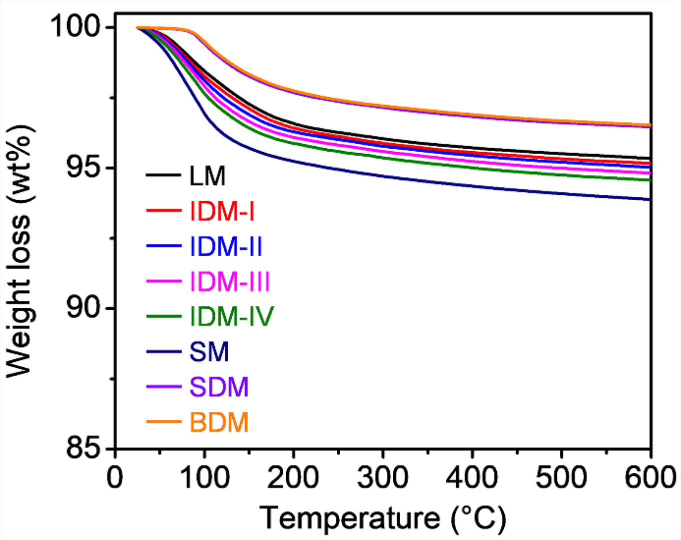

Supplementary Figure 14. TGA curves of LM, IDM, SM, SDM, and BDM films.
The TGA tests were performed under a nitrogen atmosphere from room temperature to 600ºC with a temperature ramp rate of 10ºC min^{-1}.

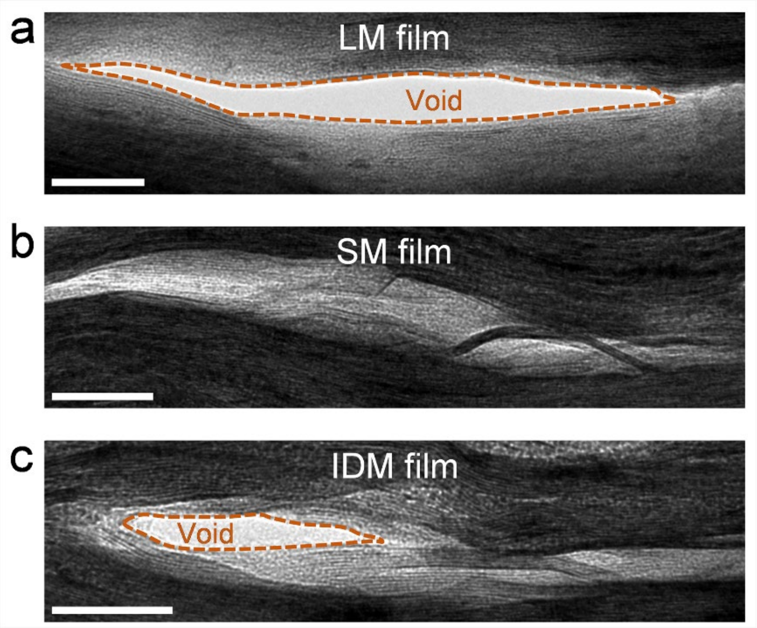

Supplementary Figure 15. TEM images of the cross-sections of films.
a, LM. **b**, SM. **c**, IDM. Scale bars, 50 nm. The LM films show oriented structure with large voids between multilayer flakes, whereas the SM films show dense structure with disordered flake stacking. The IDM films show intermediate morphology.

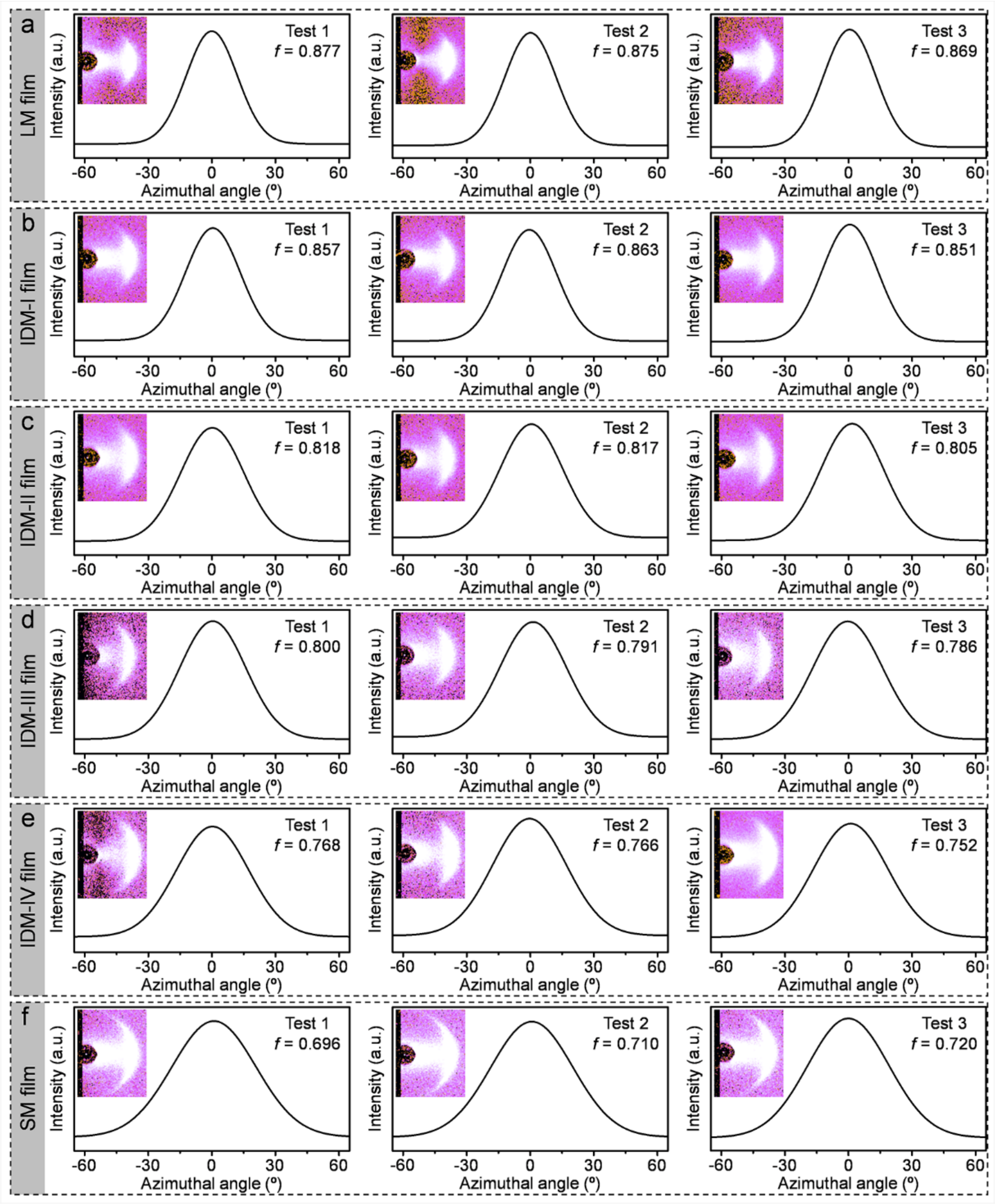

Supplementary Figure 16. WAXS patterns for an incident Cu-Kα X-ray beam parallel to the film plane and corresponding azimuthal scan profiles for the 002 peak for films.
a, LM film. **b**–**e**, IDM-I to IDM-IV films. **f**, SM film.

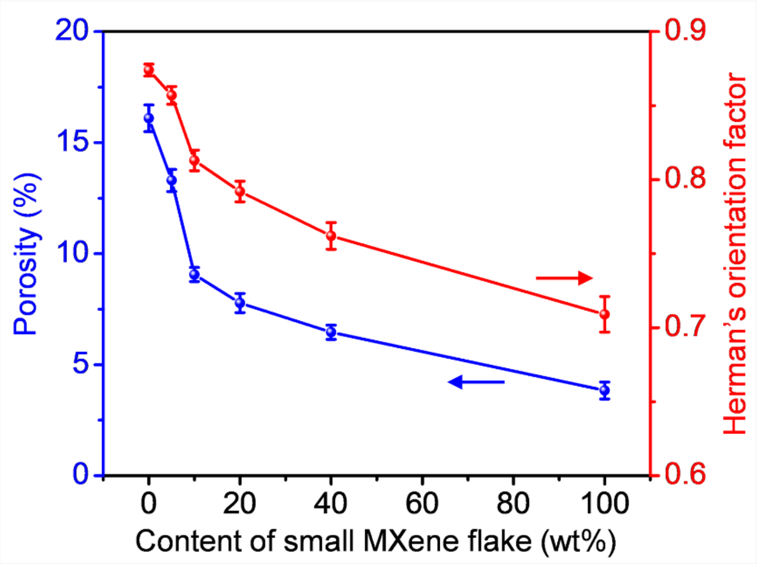

Supplementary Figure 17. Herman's orientation factor and volume percentage porosity of LM, IDM, and SM films.

The left blue ordinate is for porosity, while the right red ordinate is for Herman's orientation factor. All error bars show mean ± standard deviation.

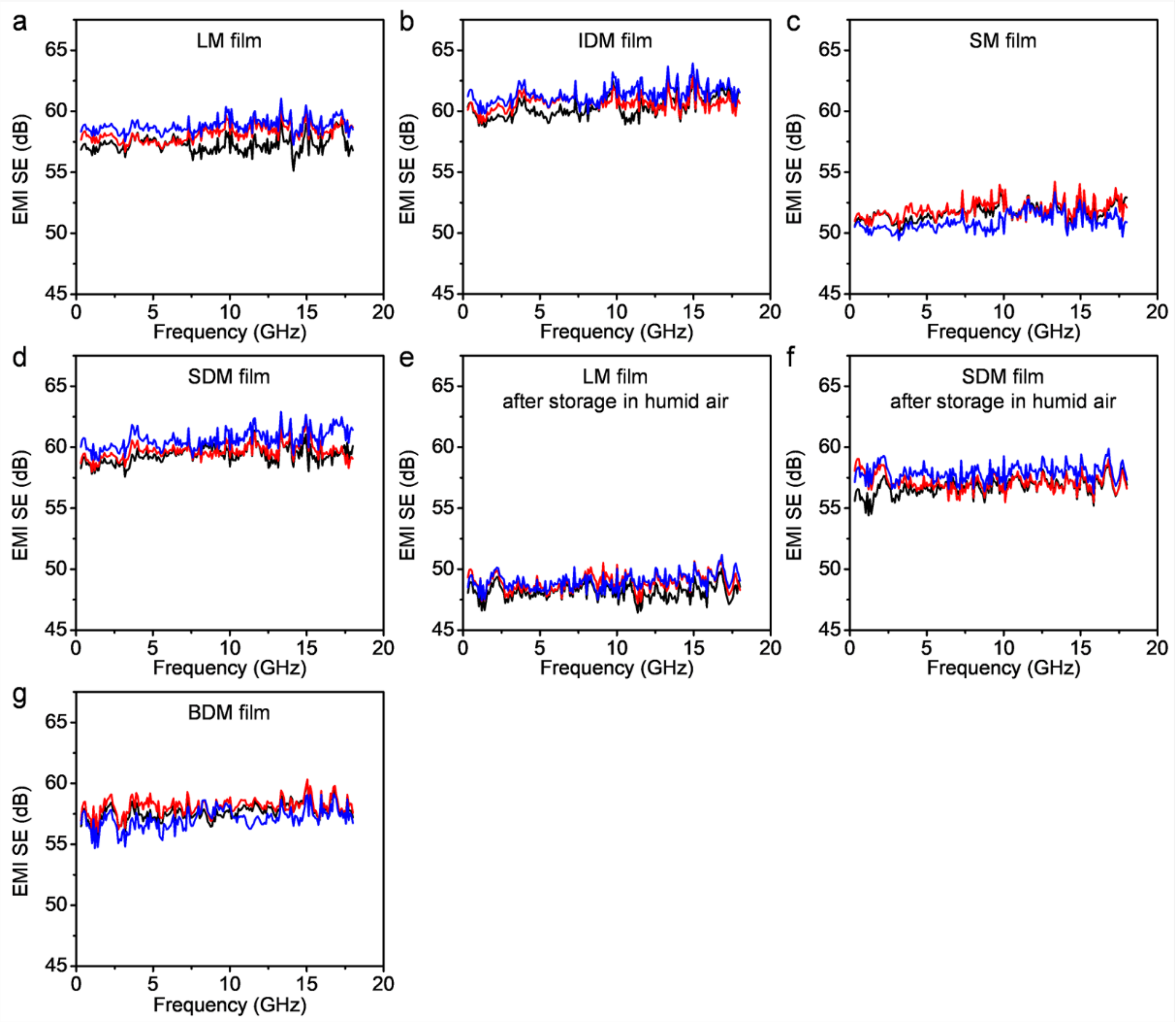

Supplementary Figure 18. EMI SE as a function of frequency for films.
a–**d**, LM (**a**), IDM (**b**), SM (**c**), and SDM (**d**) films with a thickness of 2.7 ± 0.1, 2.8 ± 0.2, 2.8 ± 0.1, and 2.5 ± 0.1 μm, respectively. **e**, **f**, LM (**e**) and SDM (**f**) films after storage in humid air for 10 days. **g**, BDM films with a thickness of 2.7 ± 0.1 μm.

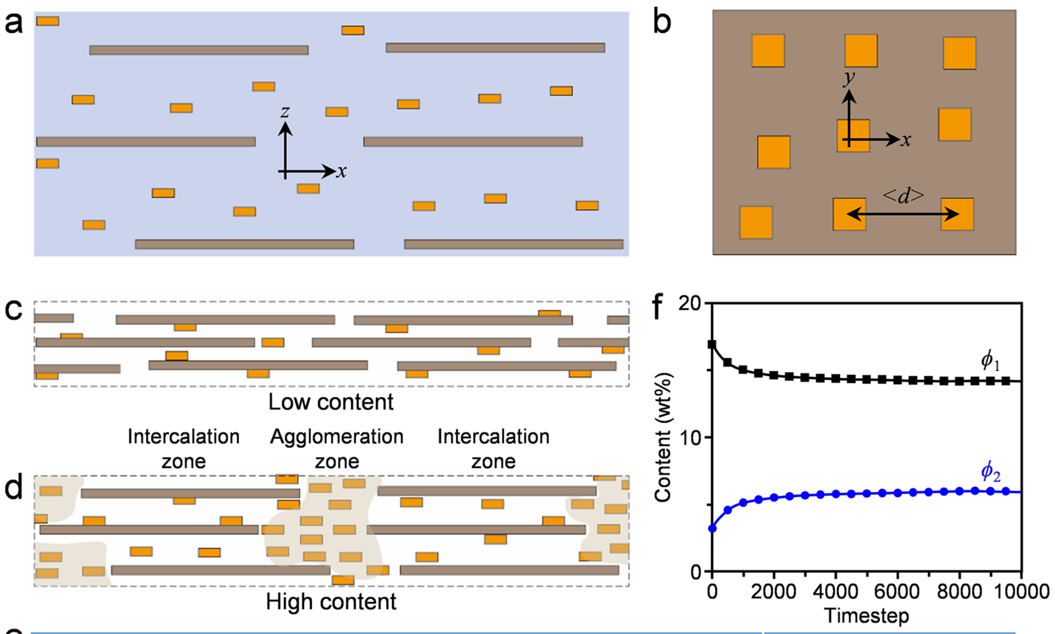

e

Variable	Value
Length of large MXene flakes, l_L	1.0
Length of small MXene flakes, l_s	0.026
Length of the gap between large flakes, l_{gap}	0.14
Maximum trial move per step, Δl_{max}	0.01
Ratio of energy barrier to thermal fluctuation energy, E_0/k_BT	2.0
Scaling constant for energy in small region, α	10.0

Supplementary Figure 19. Theoretical modeling for IDM films

a, Small MXene flakes are randomly and uniformly distributed among large ones at the initial stage before casting and drying. **b**, Translational diffusion of small flakes on a large flake. **c**, With low small flake content, the small flakes are uniformly intercalated into large MXene interlayers (denoted as the intercalation zone). **d**, With high small flake content, some small flakes agglomerate (denoted as the agglomeration zone). **e**, Dimensionless parameters used in the Monte Carlo model. **f**, Evolution of ϕ_1 and ϕ_2 simulated by the Monte Carlo method with E_0/k_BT=2.

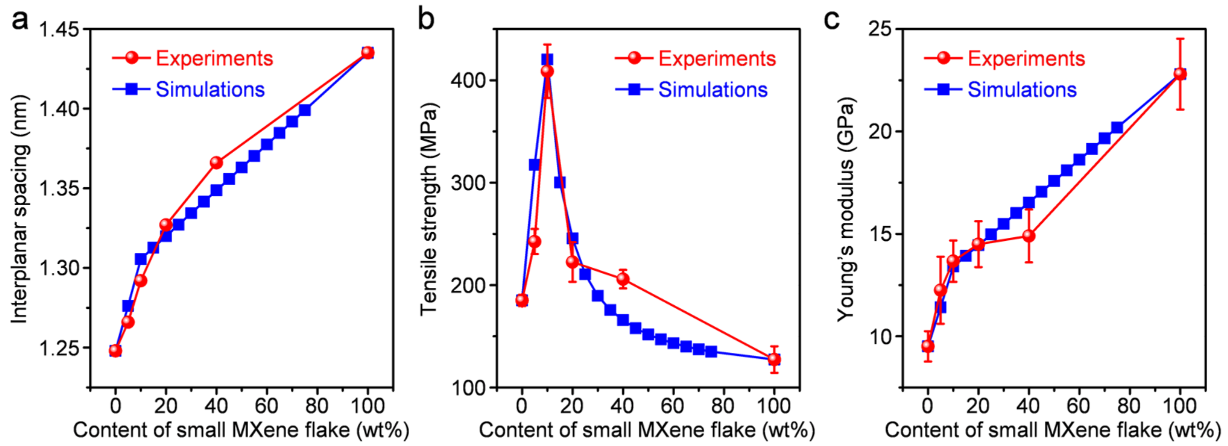

Supplementary Figure 20. Theoretical (blue square) and experimental (red circle) mechanical properties and interplanar spacing of LM, IDM, and SM films. a, Interplanar spacing. **b**, Tensile strength. **c**, Young's modulus. All error bars show mean ± standard deviation.

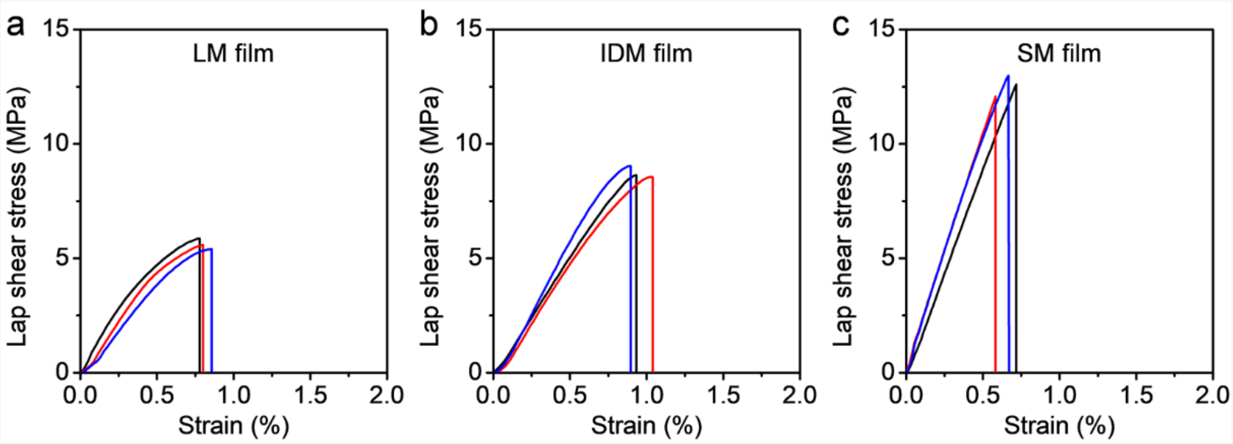

Supplementary Figure 21. Shear stress–strain curves of films. a, LM. **b**, IDM. **c**, SM.

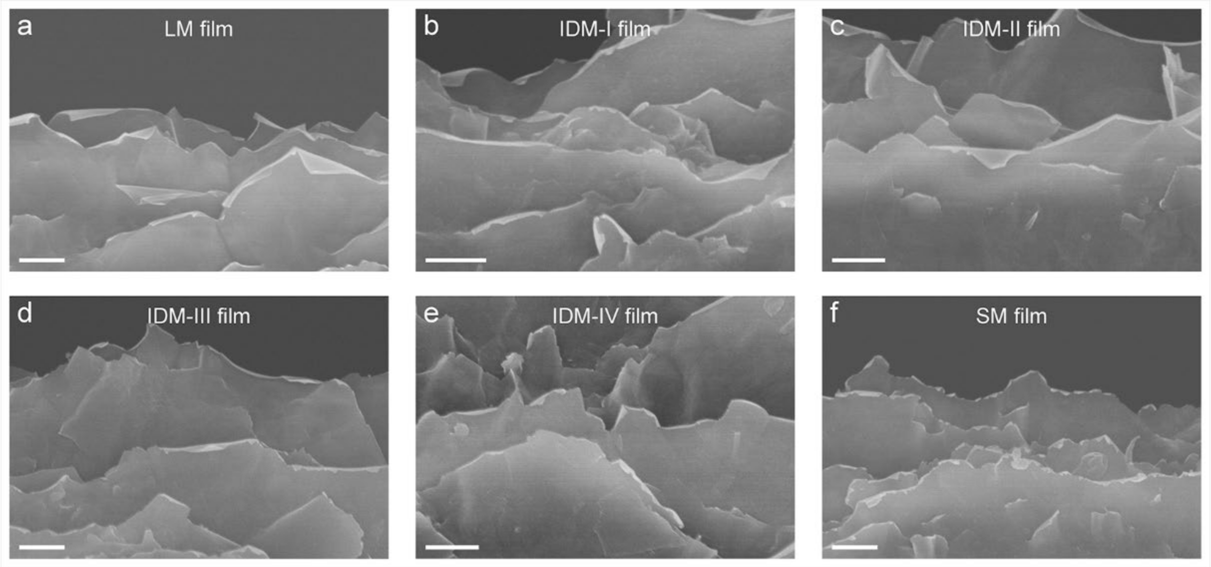

Supplementary Figure 22. Inclined-view SEM images of the tensile fracture surface of films. **a**, LM. **b**–**e**, IDM-I to IDM-IV. **f**, SM. Scale bars, 500 nm.

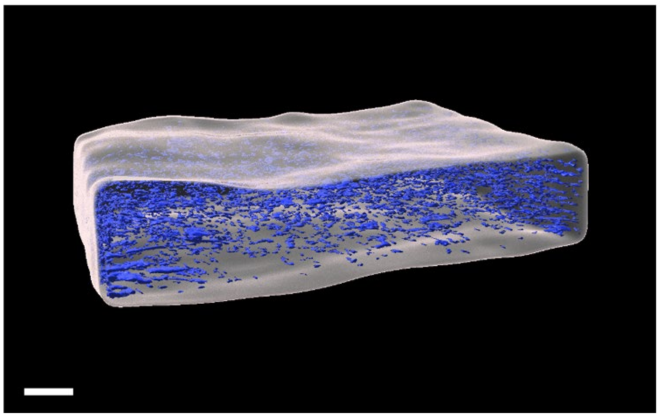

Supplementary Figure 23. 3D-reconstructed void microstructure by FIB/SEMT for BDM films. Scale bar, 2 μm.

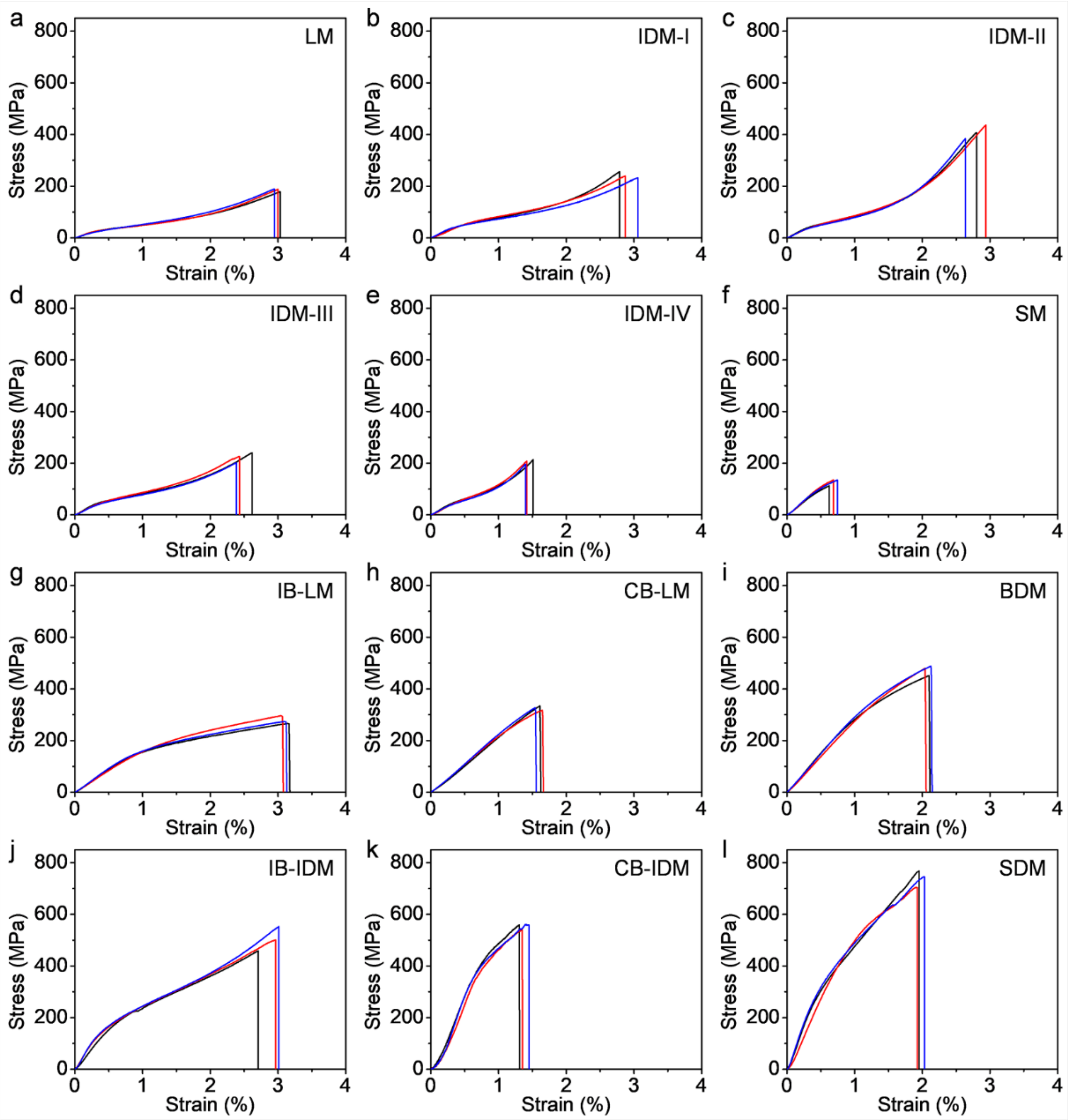

Supplementary Figure 24. Tensile stress-strain curves of films.
a, LM. **b**–**e**, IDM-I to IDM-IV. **f**, SM. **g**, IB-LM. **h**, CB-LM. **i**, BDM. **j**, IB-IDM. **k**, CB-IDM. **l**, SDM.

Supplementary Table 1. Boron and Ca^{2+} content in BDM and SDM films derived from XPS measurements.

Sample	Boron content (wt%)	Ca^{2+} content (wt%)
**BDM**	0.78	0.67
**SDM**	0.81	0.66

Supplementary Table 2. Porosity and Herman's orientation factor of LM, SM, IDM, BDM, and SDM films.

Sample	Porosity (%)	Herman's orientation factor
**LM**	16.1 ± 0.6	0.874 ± 0.004
**IDM-I**	13.3 ± 0.5	0.857 ± 0.006
**IDM-II**	9.06 ± 0.32	0.813 ± 0.007
**IDM-III**	7.78 ± 0.43	0.792 ± 0.007
**IDM-IV**	6.46 ± 0.32	0.762 ± 0.009
**SM**	3.84 ± 0.38	0.709 ± 0.012
**BDM**	9.50 ± 0.34	0.903 ± 0.006
**SDM**	4.11 ± 0.32	0.839 ± 0.004

Supplementary Table 3. Interlayer diffraction spacing of LM, SM, IDM, IB-IDM, CB-IDM, BDM, and SDM films.

Sample	*d* (nm)
**LM**	1.25
**IDM-I**	1.27
**IDM-II**	1.29
**IDM-III**	1.33
**IDM-IV**	1.37
**SM**	1.43
**IB-IDM**	1.26
**CB-IDM**	1.20
**BDM**	1.18
**SDM**	1.21

Supplementary Table 4. Thickness and mechanical properties of LM, IDM, SM, IB-LM, CB-LM, BDM, IB-IDM, CB-IDM, and SDM films.

Sample	Thickness (μm)	Young's modulus (GPa)	Tensile strength (MPa)	Toughness (MJ m^{-3})	Strain to failure (%)
**LM**	2.7 ± 0.1	9.5 ± 0.7	185 ± 6	2.36 ± 0.02	2.99 ± 0.04
**IDM-I**	2.7 ± 0.1	12.2 ± 1.6	243 ± 12	3.16 ± 0.12	2.91 ± 0.14
**IDM-II**	2.8 ± 0.2	13.7 ± 1.0	409 ± 26	4.12 ± 0.56	2.79 ± 0.15
**IDM-III**	2.6 ± 0.1	14.5 ± 1.1	223 ± 19	2.57 ± 0.30	2.48 ± 0.12
**IDM-IV**	2.7 ± 0.2	14.9 ± 1.3	206 ± 9	1.26 ± 0.13	1.44 ± 0.06
**SM**	2.8 ± 0.1	22.8 ± 1.7	127 ± 13	0.48 ± 0.10	0.68 ± 0.06
**IB-LM**	2.7 ± 0.1	17.9 ± 1.1	279 ± 16	5.65 ± 0.09	3.09 ± 0.05
**CB-LM**	2.6 ± 0.1	21.1 ± 0.4	326 ± 8	2.74 ± 0.12	1.60 ± 0.05
**BDM**	2.7 ± 0.1	29.6 ± 1.5	473 ± 19	5.71 ± 0.31	2.09 ± 0.05
**IB-IDM**	2.8 ± 0.1	43.4 ± 6.7	504 ± 47	8.40 ± 1.01	2.89 ± 0.16
**CB-IDM**	2.7 ± 0.1	65.8 ± 8.7	554 ± 11	4.41 ± 0.39	1.35 ± 0.04
**SDM**	2.5 ± 0.1	72.4 ± 8.1	739 ± 32	8.76 ± 0.52	1.96 ± 0.05

Supplementary Table 5. Electrical conductivity of LM, IDM, SM, IB-LM, CB-LM, BDM, IB-IDM, CB-IDM, and SDM films.

Sample	Electrical conductivity ($S\ cm^{-1}$)
**LM**	9,822 ± 133
**IDM-I**	10,340 ± 159
**IDM-II**	10,865 ± 203
**IDM-III**	9,998 ± 108
**IDM-IV**	8,753 ± 68
**SM**	4,837 ± 53
**IB-LM**	9,095 ± 85
**CB-LM**	9,842 ± 70
**BDM**	9,220 ± 92
**IB-IDM**	10,169 ± 112
**CB-IDM**	10,979 ± 134
**SDM**	10,336 ± 103

Supplementary Table 6. Tensile strength, electrical conductivity, and Young's modulus of SDM films and previously reported MXene films.

Number	Materials	Tensile strength (MPa)	Electrical conductivity (S cm^{-1})	Young's modulus (GPa)	Reference
**1**	**MXene**	22	2,402	3.52	18
**2**	**MXene**	23	3,300	1.4	19
**3**	**HCl-processed MXene**	102	10,400	8.8	20
**4**	**MXene**	480	10,220	18.3	21
**5**	**MXene-PEDOT:PSS**	30.2	20.4	3.1	22
**6**	**MXene-CS**	43.5	18.2	2.6	23
**7**	**MXene-PVA**	91	0.0004	3.7	18
**8**	**MXene-CNF**	135.4	0.097	3.8	24
**9**	**MXene-CNF**	341	225	42.8	25
**10**	**MXene-TOCNF**	212.2	12.1	5.9	26
**11**	**MXene-ANF**	197.1	0.295	2.01	27
**12**	**MXene-CNT**	25	130	6.25	28
**13**	**MXene-GO**	209	461.7	16.9	19
**14**	**MXene-MTM-PVA**	225	1.25	10.5	29
**15**	**MXene-Al^{3+}**	83.2	2,656	7.42	30
**16**	**MXene-SA-Ca^{2+}**	436	2,988	14.0	9
**17**	**MXene-PDA**	237	5,141	9.6	31
**18**	**MXene-PDA-Ca^{2+}**	339	4,850	12	32
**19**	**MXene-CMC-borate**	583	6,115	27.8	13
**20**	**SDM**	739	10,336	72.4	This work

Supplementary Table 7. Thickness and surface-specific SE (defined as the SE divided by thickness and density) of SDM films and other solid shielding films, including metal foils, graphene, carbon multiwalled nanotube (MWNT), carbon black (CB), and MXene composites.

Number	Materials	Thickness (mm)	surface-specific SE (dB cm^2 g^{-1})	Reference
**1**	**rGO-PS**	2.5	692	33
**2**	**rGO-Fe_3O_4**	0.3	1,033	34
**3**	**rGO-PEDOT**	0.8	841	35
**4**	**rGO-Fe_3O_4-PVA**	0.3	667	36
**5**	**Flexible graphite**	3.1	381	37
**6**	**EG-LGE**	0.043	611	38
**7**	**rGO-PCO-PSE-AP**	0.0033	39,526	39
**8**	**rGO-Cr^{3+}-PSE-AP**	0.0034	28,011	40
**9**	**rGO-BPDD**	0.0034	53,409	4
**10**	**MWNT-PC**	2.1	164	41
**11**	**MWNT-ABS**	1.1	433	42
**12**	**MWNT-PS**	2	285	43
**13**	**MWNT-WPU**	0.32	3,408	44
**14**	**MWNT-MCMB**	0.6	3,583	45
**15**	**CB-ABS**	1.1	190	42
**16**	**CB-EPDM**	2	15	46
**17**	**Copper**	3.1	32	47
**18**	**Stainless steel**	4	28	47
**19**	**Ni fiber-PES**	2.85	109	47
**20**	**Ni filaments-PES**	2.85	165	47
**21**	**Al foil**	0.008	30,555	48
**22**	**Cu foil**	0.01	7,812	48
**23**	**MXene**	0.011	25,863	48
**24**	**MXene-SA**	0.008	30,830	48

**25**	**Blade-coated MXene**	0.0024	50,872	21
**26**	**MXene-CNF**	0.047	2,647	24
**27**	**MXene-PEDOT:PSS**	0.011	19,498	22
**28**	**MXene-CA**	0.026	17,586	49
**29**	**HCl-processed MXene**	0.003	51,624	20
**30**	**MXene-CNT**	0.00017	58,187	28
**31**	**MXene-TOCNF**	0.047	4,761	26
**32**	**MXene-MTM-PVA**	0.003	24,550	29
**33**	**MXene-ANF**	0.017	13,176	27
**34**	**MXene-SA-Ca^{2+}**	0.0028	58,929	9
**35**	**MXene-PDA**	0.00695	26,096	31
**36**	**MXene-CMC-borate**	0.003	62,458	13
**37**	**SDM**	0.0025	71,801	This work

Supplementary References

1. Park, H. et al. Dynamic assembly of liquid crystalline graphene oxide gel fibers for ion transport. *Sci. Adv.* **4**, eaau2104 (2018).
2. Ding, L. et al. MXene molecular sieving membranes for highly efficient gas separation. *Nat. Commun.* **9**, 155 (2018).
3. Naguib, M., Mochalin, V. N., Barsoum, M. W. & Gogotsi, Y. 25th anniversary article: MXenes: a new family of two-dimensional materials. *Adv. Mater.* **26**, 992-1005 (2014).
4. Wan, S. et al. Ultrastrong graphene films via long-chain π-bridging. *Matter* **1**, 389-401 (2019).
5. Kim, S. G., Wang, S. H., Ok, C. M., Jeong, S. Y. & Lee, H. S. Lateral diffusion of graphene oxides in water and the size effect on the orientation of dispersions and electrical conductivity. *Carbon* **125**, 280-288 (2017).
6. Wang, X. et al. Atomic-scale recognition of surface structure and intercalation mechanism of Ti_3C_2X. *J. Am. Chem. Soc.* **137**, 2715-2721 (2015).
7. Metropolis, N., Rosenbluth, A. W., Rosenbluth, M. N. & Teller, A. H. Equation of state calculations by fast computing machines. *J. Chem. Phys.* **21**, 1087-1092 (1953).
8. Shen, J. et al. 2D MXene nanofilms with tunable gas transport channels. *Adv. Funct. Mater.* **28**, 1801511 (2018).
9. Wan, S. et al. Strong sequentially bridged MXene sheets. *Proc. Natl. Acad. Sci. U.S.A.* **117**, 27154-27161 (2020).
10. Zhang, C. J. et al. Oxidation stability of colloidal two-dimensional titanium carbides (MXenes). *Chem. Mater.* **29**, 4848-4856 (2017).
11. Lipatov, A. et al. Elastic properties of 2D $Ti_3C_2T_x$ MXene monolayers and bilayers. *Sci. Adv.* **4**, eaat0491 (2018).
12. Ren, C. E. et al. Charge- and size-selective ion sieving through $Ti_3C_2T_x$ MXene membranes. *J. Phys. Chem. Lett.* **6**, 4026-4031 (2015).
13. Wan, S. et al. High-strength scalable MXene films through bridging-induced densification. *Science* **374**, 96-99 (2021).
14. Iqbal, A. et al. Anomalous absorption of electromagnetic waves by 2D transition metal carbonitride Ti_3CNT_x (MXene). *Science* **369**, 446-450 (2020).
15. Shi, X. et al. Bioinspired ultrasensitive and stretchable MXene-based strain sensor via nacre-mimetic microscale "brick-and-mortar" architecture. *ACS Nano* **13**, 649-659 (2019).
16. An, Z., Compton, O. C., Putz, K. W., Brinson, L. C. & Nguyen, S. T. Bio-inspired borate cross-linking in ultra-stiff graphene oxide thin films. *Adv. Mater.* **23**, 3842-3846 (2011).
17. Shahzadi, K. et al. Bio-based artificial nacre with excellent mechanical and barrier properties realized by a facile *in situ* reduction and cross-linking reaction. *ACS Nano* **11**, 325-334 (2017).
18. Ling, Z. et al. Flexible and conductive MXene films and nanocomposites with high capacitance. *Proc. Natl. Acad. Sci. U.S.A.* **111**, 16676-16681 (2014).
19. Liu, J. et al. Ultrastrong and highly conductive MXene-based films for high-performance electromagnetic interference shielding. *Adv. Electron. Mater.* **6**, 1901094 (2020).
20. Chen, H. et al. Pristine titanium carbide MXene films with environmentally stable conductivity and superior mechanical strength. *Adv. Funct. Mater.* **30**, 1906996 (2020).
21. Zhang, J. et al. Scalable manufacturing of free-standing, strong $Ti_3C_2T_x$ MXene films with

outstanding conductivity. *Adv. Mater.* **32**, 2001093 (2020).
22. Liu, R. et al. Ultrathin biomimetic polymeric $Ti_3C_2T_x$ MXene composite films for electromagnetic interference shielding. *ACS Appl. Mater. Interfaces* **10**, 44787-44795 (2018).
23. Hu, C. et al. Characteristics of Ti_3C_2X-chitosan films with enhanced mechanical properties. *Front. Energy Res.* **4**, 41 (2017).
24. Cao, W. T. et al. Binary strengthening and toughening of MXene/cellulose nanofiber composite paper with nacre-inspired structure and superior electromagnetic interference shielding properties. *ACS Nano* **12**, 4583-4593 (2018).
25. Tian, W. et al. Multifunctional nanocomposites with high strength and capacitance using 2D MXene and 1D nanocellulose. *Adv. Mater.* **31**, 1902977 (2019).
26. Zhan, Z., Song, Q., Zhou, Z. & Lu, C. Ultrastrong and conductive MXene/cellulose nanofiber films enhanced by hierarchical nano-architecture and interfacial interaction for flexible electromagnetic interference shielding. *J. Mater. Chem. C* **7**, 9820-9829 (2019).
27. Xie, F. et al. Ultrathin MXene/aramid nanofiber composite paper with excellent mechanical properties for efficient electromagnetic interference shielding. *Nanoscale* **11**, 23382-23391 (2019).
28. Weng, G. M. et al. Layer-by-layer assembly of cross-functional semi-transparent MXene-carbon nanotubes composite films for next-generation electromagnetic interference shielding. *Adv. Funct. Mater.* **28**, 1803360 (2018).
29. Lipton, J. et al. Mechanically strong and electrically conductive multilayer MXene nanocomposites. *Nanoscale* **11**, 20295-20300 (2019).
30. Liu, Z. et al. Electrically conductive aluminum ion-reinforced MXene films for efficient electromagnetic interference shielding. *J. Mater. Chem. C* **8**, 1673-1678 (2020).
31. Lee, G. S. et al. Mussel inspired highly aligned $Ti_3C_2T_x$ MXene film with synergistic enhancement of mechanical strength and ambient stability. *ACS Nano* **14**, 11722-11732 (2020).
32. Usman, K. A. S. et al. Sequentially bridged $Ti_3C_2T_x$ MXene sheets for high performance applications. *Adv. Mater. Interfaces* **8**, 2002043 (2021).
33. Yan, D.-X. et al. Structured reduced graphene oxide/polymer composites for ultra-efficient electromagnetic interference shielding. *Adv. Funct. Mater.* **25**, 559-566 (2015).
34. Song, W.-L. et al. Magnetic and conductive graphene papers toward thin layers of effective electromagnetic shielding. *J. Mater. Chem. A* **3**, 2097-2107 (2015).
35. Agnihotri, N., Chakrabarti, K. & De, A. Highly efficient electromagnetic interference shielding using graphite nanoplatelet/poly(3,4-ethylenedioxythiophene)–poly(styrenesulfonate) composites with enhanced thermal conductivity. *RSC Adv.* **5**, 43765-43771 (2015).
36. Bhaskara Rao, B. V. et al. Single-layer graphene-assembled 3D porous carbon composites with PVA and Fe_3O_4 nano-fillers: an interface-mediated superior dielectric and EMI shielding performance. *Phys. Chem. Chem. Phys.* **17**, 18353-18363 (2015).
37. Luo, X. & Chung, D. D. L. Electromagnetic interference shielding reaching 130 dB using flexible graphite. *Carbon* **34**, 1293-1294 (1996).
38. Liu, Y. et al. Graphene enhanced flexible expanded graphite film with high electric, thermal conductivities and EMI shielding at low content. *Carbon* **133**, 435-445 (2018).
39. Wan, S. et al. Sequentially bridged graphene sheets with high strength, toughness, and

electrical conductivity. *Proc. Natl. Acad. Sci. U.S.A.* **115**, 5359-5364 (2018).

40. Wan, S., Fang, S., Jiang, L., Cheng, Q. & Baughman, R. H. Strong, conductive, foldable graphene sheets by sequential ionic and π bridging. *Adv. Mater.* **30**, 1802733 (2018).
41. Pande, S., Chaudhary, A., Patel, D., Singh, B. P. & Mathur, R. B. Mechanical and electrical properties of multiwall carbon nanotube/polycarbonate composites for electrostatic discharge and electromagnetic interference shielding applications. *RSC Adv.* **4**, 13839-13849 (2014).
42. Al-Saleh, M. H., Saadeh, W. H. & Sundararaj, U. EMI shielding effectiveness of carbon based nanostructured polymeric materials: a comparative study. *Carbon* **60**, 146-156 (2013).
43. Arjmand, M., Apperley, T., Okoniewski, M. & Sundararaj, U. Comparative study of electromagnetic interference shielding properties of injection molded versus compression molded multi-walled carbon nanotube/polystyrene composites. *Carbon* **50**, 5126-5134 (2012).
44. Zeng, Z. et al. Thin and flexible multi-walled carbon nanotube/waterborne polyurethane composites with high-performance electromagnetic interference shielding. *Carbon* **96**, 768-777 (2016).
45. Chaudhary, A. et al. Lightweight and easily foldable MCMB-MWCNTs composite paper with exceptional electromagnetic interference shielding. *ACS Appl. Mater. Interfaces* **8**, 10600-10608 (2016).
46. Ghosh, P. & Chakrabarti, A. Conducting carbon black filled EPDM vulcanizates: assessment of dependence of physical and mechanical properties and conducting character on variation of filler loading. *Eur. Polym. J.* **36**, 1043-1054 (2000).
47. Shui, X. & Chung, D. D. L. Nickel filament polymer-matrix composites with low surface impedance and high electromagnetic interference shielding effectiveness. *J. Electron. Mater.* **26**, 928-934 (1997).
48. Shahzad, F. et al. Electromagnetic interference shielding with 2D transition metal carbides (MXenes). *Science* **353**, 1137-1140 (2016).
49. Zhou, Z. et al. Ultrathin MXene/calcium alginate aerogel film for high-performance electromagnetic interference shielding. *Adv. Mater. Interfaces* **6**, 1802040 (2019).
