# Supplementary material for: Ultrastrong MXene films via the synergy of intercalating small flakes and interfacial bridging
Source: Nat Commun. 2022 Nov 29;13:7340. doi: 10.1038/s41467-022-35226-0 (PMC9708659; doi:10.1038/s41467-022-35226-0)
Supplement: Supplementary file 2 — Description of Additional Supplementary Files [file 41467_2022_35226_MOESM2_ESM.pdf]

Description of Additional Supplementary Files

File Name: Supplementary Movie 1
Description: A series of sections derived from FIB/SEMT for an about 9.36×17.3 μm^2 region of SDM films. A cross-section image containing the scale bar is shown in Supplementary Fig. 7d. These sections derived from FIB/SEMT are not perpendicular to the film surface.

File Name: Supplementary Movie 2
Description: 3D observation of reconstructed void microstructure derived from FIB/SEMT for an about 9.36×17.3 μm^2 region of SDM films. The film regions in Supplementary Movies 1 and 2 are identical. A movie snapshot containing the scale bar is shown in Fig. 1d.

File Name: Supplementary Movie 3
Description: A series of sections derived from FIB/SEMT for an about 9.57×15.8 μm^2 region of LM films. A cross-section image containing the scale bar is shown in Supplementary Fig. 7a. These sections derived from FIB/SEMT are not perpendicular to the film surface.

File Name: Supplementary Movie 4
Description: 3D observation of reconstructed void microstructure derived from FIB/SEMT for an about 9.57×15.8 μm^2 region of LM films. The film regions in Supplementary Movies 3 and 4 are identical. A movie snapshot containing the scale bar is shown in Fig. 2b.

File Name: Supplementary Movie 5
Description: A series of sections derived from FIB/SEMT for an about 9.00×16.2 μm^2 region of SM films. A cross-section image containing the scale bar is shown in Supplementary Fig. 7b. These sections derived from FIB/SEMT are not perpendicular to the film surface.

File Name: Supplementary Movie 6
Description: 3D observation of reconstructed void microstructure derived from FIB/SEMT for an about 9.00×16.2 μm^2 region of SM films. The film regions in Supplementary Movies 5 and 6 are identical. A movie snapshot containing the scale bar is shown in Fig. 2e.

File Name: Supplementary Movie 7
Description: A series of sections derived from FIB/SEMT for an about 9.48×15.4 μm^2 region of IDM films. A cross-section image containing the scale bar is shown in Supplementary Fig. 7c. These sections derived from FIB/SEMT are not perpendicular to the film surface.

File Name: Supplementary Movie 8
Description: 3D observation of reconstructed void microstructure derived from FIB/SEMT for an about 9.48×15.4 μm^2 region of IDM films. The film regions in Supplementary Movies 7 and 8 are identical. A movie snapshot containing the scale bar is shown in Fig. 2h.

File Name: Supplementary Movie 9
Description: A series of sections derived from FIB/SEMT for an about 9.57×15.7 μm^2 region of BDM films. A cross-section image containing the scale bar is shown in Supplementary Fig. 7e. These sections derived from FIB/SEMT are not perpendicular to the film surface.

File Name: Supplementary Movie 10
Description: 3D observation of reconstructed void microstructure derived from FIB/SEMT for an about 9.57×15.7 μm^2 region of BDM films. The film regions in Supplementary Movies 9 and 10 are identical. A movie snapshot containing the scale bar is shown in Supplementary Fig. 23.
